# Building trauma-informed and equitable capacity for city programs: enhancing workforce well-being and service quality in Boston

**DOI:** 10.3389/fpubh.2026.1777682

**Published:** 2026-05-29

**Authors:** Krupa Boradia, Kimberly Mendoza Iraheta, Michaela Harris Harrell, Shanika Bourne, Krystal Abbott, Samara Grossman, Bronwen White

**Affiliations:** 1 New York University, New York, NY, United States; 2 Boston Public Health Commission, Boston, MA, United States; 3 Baker Center for Children and Families, Harvard Medical School, Boston, MA, United States

**Keywords:** health equity, Learning Collaborative, quality improvement, resilience, trauma, trauma-informed, vicarious trauma, workforce well-being

## Abstract

This manuscript presents the outcomes of a mixed-methods case study evaluating the implementation of a Trauma-Informed and Equitable (TIE) Learning Collaborative within the City of Boston workforce. It seeks to move beyond the theoretical benefits of trauma-informed care (TIC) to explore potential implementation drivers within real world settings that may predict TIC’s successful program integration, focusing on readiness and capacity, acceptability, and self-care practices. Our findings reveal the complexities of implementing multi-level change initiatives within this sector, particularly during a period of increased macro-level stressors facing the public workforce. This study explores the gap between evidence-based interventions and their practical operationalization and evaluation in novel contexts. It provides an exploratory implementation study of strategies focusing on this underserved workforce and their constituents, the successes and challenges of which may offer useful insights for other practitioners and researchers navigating similar complexities.

## 1 Introduction

Communities across the United States are increasingly recognizing the profound impact of trauma and systemic inequities on public health outcomes. Addressing these challenges demands more than isolated, individual interventions. It instead requires a coordinated, multi-level approach that equips organizations with the tools to foster resilience, advance equity, and sustain meaningful changes over time for both workers and those they serve.

This case study will cover the rationale, process, and outcomes of the City Learning Collaborative. We will provide a model describing hypothesized causal pathways for how our intervention intended to promote workforce well-being and trauma-informed and equitable services, including key components, implementation drivers, and evaluation strategies. We outline how our Learning Collaborative is based on a quality improvement model which was strategically adapted to support essential City of Boston programs (a novel application). We explain how the process and structure of the Collaborative sought to engage leadership and direct care staff in developing changes within their programs. Finally, we describe our evaluation process, limitations and preliminary findings of the pilot City Collaborative, including promising outcomes, important challenges, and recommendations for future implementation.

The hope of the authors is that this case study can bridge the “practice to science” cycle (1) by offering important lessons for other municipalities who may also be seeking to strengthen resident services and workforce well-being in the wake of (or amidst ongoing) collective crisis.

## 2 Context

### 2.1 Understanding trauma, vicarious trauma, and resilience

Trauma encompasses experiences that are physically or emotionally harmful or life-threatening, resulting in lasting negative impacts on an individual’s mental, physical, emotional, social, or spiritual well-being (2). Trauma occurs on multiple levels and often intersects. Interpersonal trauma includes adverse childhood experiences (ACEs) such as abuse, neglect, and household dysfunction (3). ACEs are alarmingly common: over 60% of U. S. adults report at least one ACE, and prevalence is notably higher among communities impacted by systemic racism, poverty, and structural inequities (4). In addition to exposure to multiple forms of violence, such as sexual, domestic, and community violence, individuals are impacted by structural trauma, which includes housing instability, food insecurity, and institutional discrimination, that together can perpetuate intergenerational harm (5, 6).

Another domain of trauma includes vicarious trauma. In 2013, updates to the DSM-5 included for the first time trauma exposure in a professional capacity as meeting the criteria for a traumatic stressor (7). This expansion recognizes the way in which service providers working with those who have experienced trauma may themselves also be impacted negatively “through repeated exposure and empathic engagement with their clients’ and patients’ traumatic experiences” (8–11). In addition to impacting the well-being of the worker, vicarious trauma can have negative impacts on service quality as a result of provider turnover, symptom interference in work performance, increased absences, and reduced productivity (12, 13).

Without intervention, traumatic stress has well-documented, long-term health impacts including higher rates of heart disease, cancer, substance use, and mental illness (3). However, protective factors such as access to safe, stable, nurturing adults, housing, food, mental healthcare and community cohesion all play a role in buffering the impacts of trauma and fostering resilience (14). In the workplace, both individual and organizational factors influence worker well-being and resilience. These include individual’s positive coping strategies (15) and social support (16) and organizational factors such as physical and psychological safety (17–23), a sense of belonging (24–28), quality supervision (29–32), training and opportunities for advancement (33–39), and fair wages, time off and other benefits (40–51).

The concept of resilience faces important criticism due to how it can be used to focus on the role of “individual responsibility” and capacity to “bounce back” from trauma and adversity, rather than addressing the underlying social and structural determinants causing harm (52).

Another conceptualization of resilience is that of individual and collective capacities to survive, adapt, resist, and find meaning despite hardship and adversity. Resilience itself is shaped by our families, communities, culture, systems, and institutions. Additionally, promoting resilience must happen through ensuring access to rights, resources, and representation (CBTI, Boston Public Health Commission, unpublished data, 2024).

### 2.2 Availability of funding to address trauma through workforce development

Massachusetts was among the first states to report COVID-19 cases and had the nation’s third-highest mortality rate until fall 2021 (53). The COVID-19 pandemic amplified the Commonwealth’s existing workforce challenges, such as trauma exposure and retention, with direct implications for service quality. In response, the American Rescue Plan Act (ARPA) allocated funding to the City of Boston to mitigate the public and mental health impacts of the pandemic, including through the Boston Public Health Commission’s (BPHC) Center for Behavioral Health and Wellness.

In addition to the BPHC and the Center’s other pandemic recovery efforts, the BPHC’s Capacity Building and Training Initiative (CBTI) program received ARPA funding to mitigate impacts on the City of Boston public sector workforce. This is due to CBTI’s specialization in developing trauma-informed and equitable systems of care via workforce development initiatives. One of these initiatives is the City Learning Collaborative and the focus of this case study.

### 2.3 Intervention strategy development

Recognizing the widespread and often invisible nature of trauma and the importance of fostering a nuanced conceptualization of resilience, one option for practitioners to respond is to utilize a trauma-informed care (TIC) approach. TIC transforms the inquiry from “What is wrong with you?” to “What happened to you?” The Substance Abuse and Mental Health Services Administration (SAMHSA) offers six principles for a trauma-informed care framework that organizations and service providers can integrate in their daily practice with those they serve: safety, trustworthiness and transparency, peer support, collaboration, empowerment, and attention to cultural, historical, and gender issues (2, 54).

**Figure 1:**
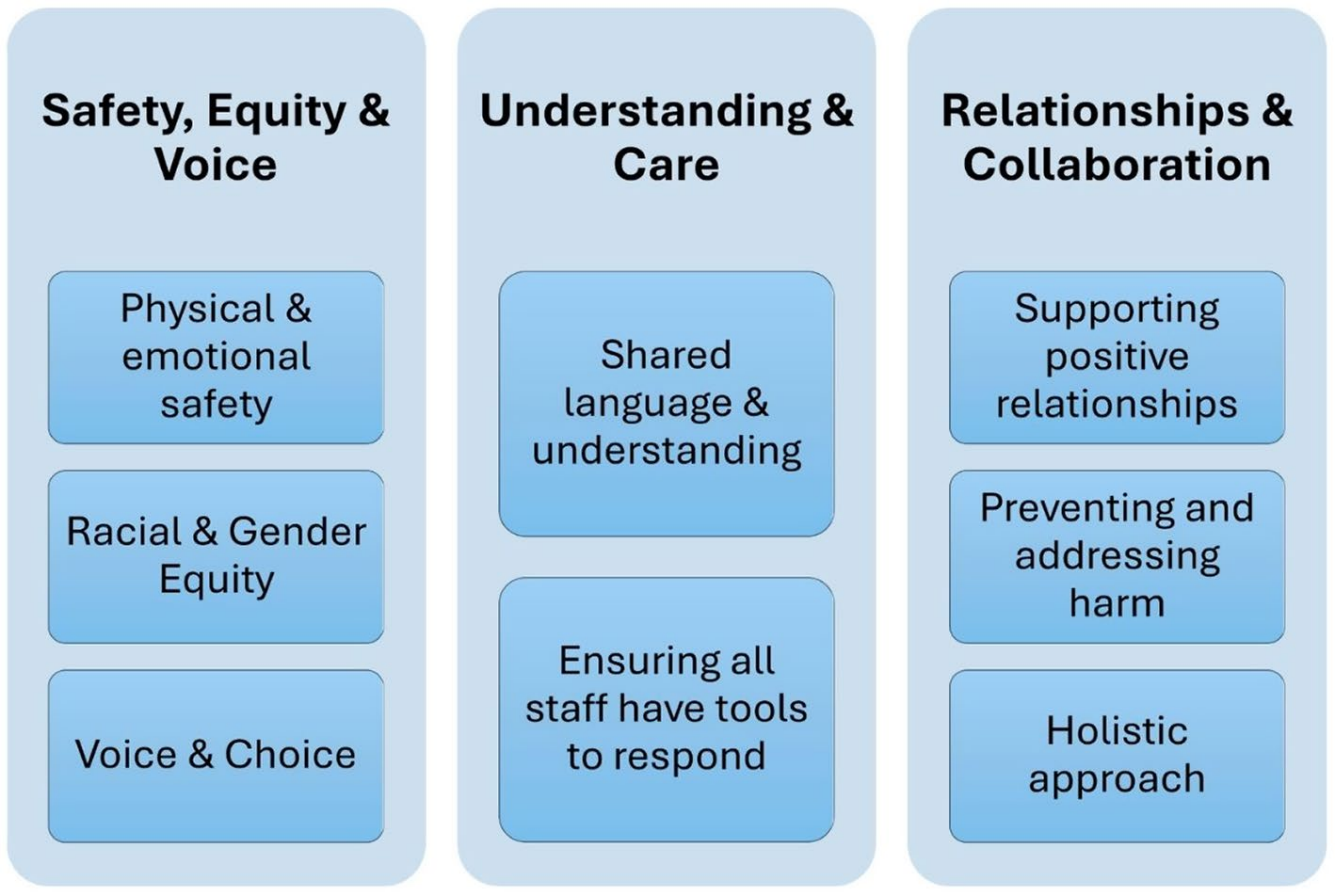
Trauma-informed and equitable principles utilized by implementation team (Capacity Building & Training Initiative, Boston Public Health Commission).

Since 2013 the CBTI team used an iterative approach to adapt SAMHSA’s principles of trauma-informed care to meet the local context. This includes ongoing review of the literature and feedback from over 8,000 training participants, such as youth workers, family advocates, violence responders, and other direct care providers, informing their evolution to their current form. See Figure 1 for CBTI’s adapted Trauma-Informed and Equitable (TIE) Principles.

CBTI elected to use a Learning Collaborative model to support City programs in implementing trauma-informed and equitable care in their respective contexts. This was due to both CBTI program capacity and the evidence base for Learning Collaboratives as an effective quality improvement model.

Originally developed in healthcare to improve patient outcomes (55), Learning Collaboratives bridge research and practice through:

- Structured cycles of learning, implementation, and evaluation.
- Peer-driven problem-solving, uniting interdisciplinary teams.
- Sustained engagement to drive lasting change (56).

Success in other sectors, such as behavioral health (57) and education (58, 59), informed CBTI’s adaptation for municipal settings—a novel application.

## 3 Key programmatic elements and findings

### 3.1 Overall goal and conceptual model

**Figure 2:**
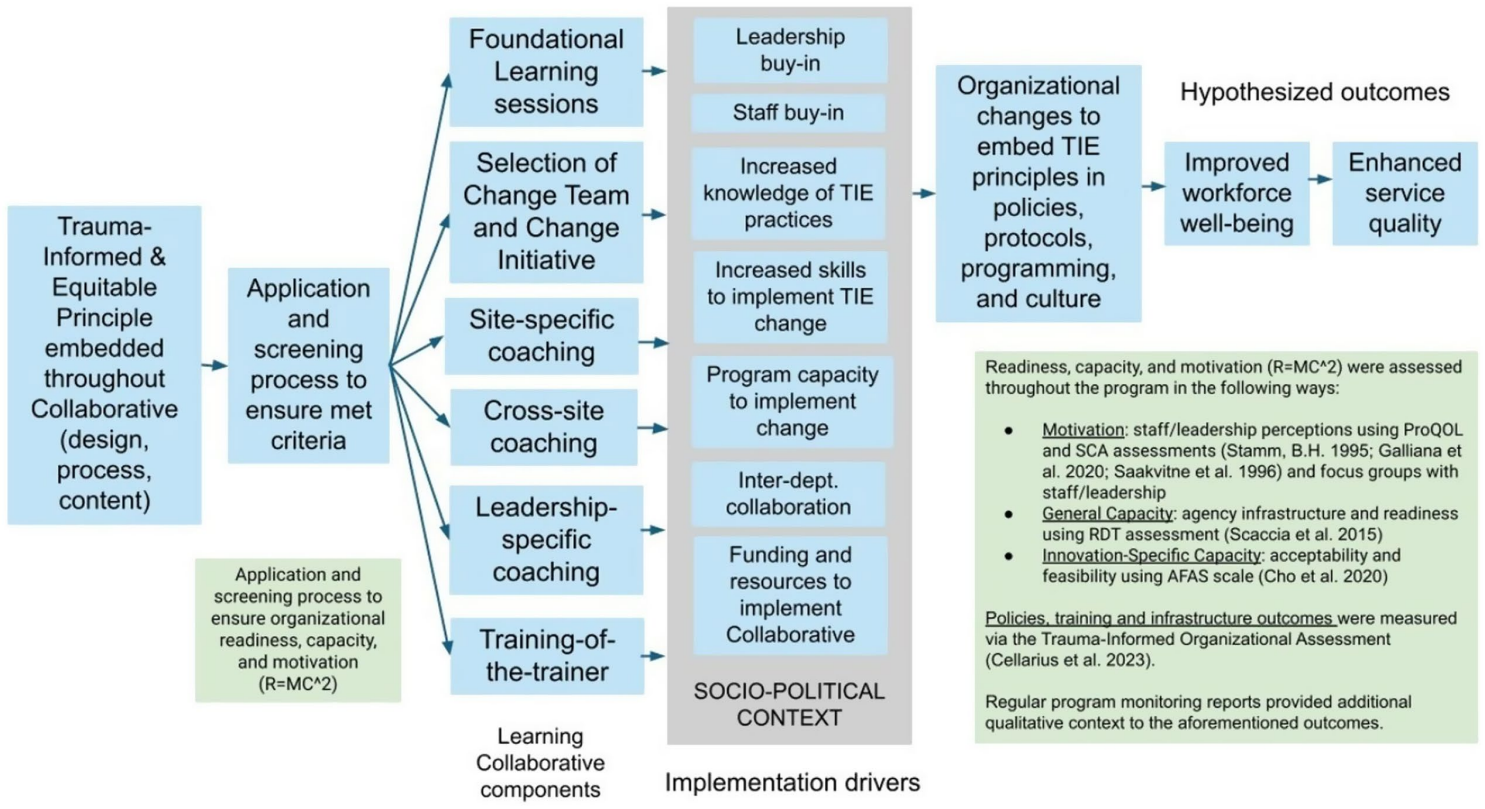
Model of City Learning Collaborative theorized causal pathways.

The overall goal of the initiative was to utilize a Learning Collaborative model to support at least five City of Boston programs in integrating trauma-informed and equitable principles into their policies, practices, culture and/or procedures, with the assumption being such integration will result in long-term improvements in workforce well-being and service quality. For a visualization of the components of the intervention and hypothesized causal pathways, see a conceptual model in Figure 2.

### 3.2 Evaluation design

To assess the implementation and early outcomes of the City Learning Collaborative (“the Collaborative), the evaluation was guided by two central questions (1) Was the Learning Collaborative implemented as intended? And (2) were the intended outcomes achieved?

To address these questions, the evaluation examined the following aims:

- Implementation and participation: to assess whether the Collaborative was implemented as designed, including participation across agencies and engagement in core activities.
- Organizational readiness and capacity: to examine changes in organizational readiness and capacity to implement a TIE change initiative across programs.
- Workforce well-being and individual capacity: to evaluate changes in workforce well-being and individual level practices, including staff self-care practices, burnout, compassion satisfaction, and confidence in applying TIE skills.
- Implementation facilitators and barriers: to identify contextual factors influencing implementation, including facilitators and barriers related to leadership and staff engagement, organizational priorities, and external contextual influences.

To achieve these aims the evaluators utilized a mixed-methods approach, specifically using the R = MC^2^ readiness framework. This model guided our investigation into the critical drivers of implementation: organizational Motivation (M), general Capacity (C), and innovation-specific Capacity (C), which collectively determine Readiness (R) for change (60).

Quantitative data was collected via surveys and analyzed using SPSS statistical software, while qualitative data was collected via interviews and analyzed using Dedoose software with rapid qualitative analysis (61).

Regular program monitoring reports provided additional qualitative context to these data.

### 3.3 Preparing for implementation

Partnerships between key stakeholders were crucial in the design and implementation of the Collaborative. Stakeholders included:

- Lead implementation team: Capacity Building and Training Initiative, Boston Public Health Commission (BPHC).
- Funder and oversight: Center for Behavioral Health and Wellness, BPHC
- Advisory group stakeholders: Representatives from key City cabinets and programs.
- Evaluation partner: The Baker Center

The Collaborative is being implemented in two cohorts, with the first pilot cohort being from March 2024 to June 2025, and the second from January 2026 to December 2026.

Following the receipt of funding, planning for the first cohort began in the spring of 2023, including the selection of an evaluation and training partner. It also included the convening of an advisory group of City stakeholders to inform the program’s goals, objectives, and structure, modeling a trauma-informed and equitable approach by using a participatory design process.

Given the workload associated with successful implementation, a crucial step was the hiring of a full-time employee to lead the project in the fall of 2023. The full-time employee joined the Implementation Team to finalize the Collaborative’s goals, develop a work plan for the first phase of the project, work with evaluation team to design the plan, and create a recruitment strategy.

### 3.4 Recruitment and inclusion criteria

Participants were recruited via outreach to City employee networks and advisory group members. During screening, interested leadership and staff members were interviewed individually to candidly assess readiness for participation and change.

Programs were selected based on:

1. Representation: ≥2 staff per program (senior leadership and direct care staff).
2. Commitment: Interest in gaining knowledge of trauma-informed & equitable practices, dedication to workforce well-being, and willingness for organizational change by both staff and leadership.
3. Capacity: Ability to participate in mandatory foundational learning sessions, coaching sessions, evaluation activities and adoption of at least one TIE-aligned change to policies, programming, and/or culture during the program. Participants were also required to monitor and report on progress through an Implementation Work Plan and communication with the CBTI team. Participants were also informed of the optional Training-of-Trainer (TOT) sessions.

### 3.5 Implementation

As indicated in Figure 2, the Collaborative involved multiple components, including: Foundational Learning sessions, program identification of Change Teams and Change Initiatives, Site-Specific Coaching, Cross-Site Coaching, Leadership-Specific Coaching, and a Training-of-the-Trainer (TOT) series.

The CBTI team adapted much of the Foundational Learning content from CBTI’s existing curricula on trauma, resilience, and equity. CBTI and the evaluation team co-developed the coaching framework and curriculum from both CBTI’s existing content and additional quality improvement literature and tools.

Following foundational learning, each program was asked to form their Change Team. Change Teams were tasked with identifying their Change Initiative, which is defined as any deliberate intervention or action that aims to help individuals, teams, or organizations grow, transform, or improve using trauma-informed and equitable principles (62). A Readiness and Goal Setting Tool and Prioritization Matrix tool were used during the first coaching session to identify program’s top three priorities for change, existing challenges and strengths, and ultimately, select one change initiative.

Subsequent coaching sessions focused on providing guidance on change implementation, including navigating challenges related to buy-in among the broader program staff and leadership, effective activity design, and bolstering team capacity.

The TOT was offered as an optional opportunity, with 15 participants from cross-organizational teams including individuals from programs that participated in the Collaborative and individuals that were part of programs not previously involved in the Collaborative. The purpose of the TOT was to enhance internal capacity and promote sustainability by equipping participants with tools and strategies for trauma-informed and equitable facilitation in general and on trauma, resilience, and equity content specifically.

Upon completion of cohort 1, completion certificates were awarded to participants. Certificates are of interest to direct care staff for use as documentation of participation in professional development when seeking career advancement opportunities.

Three programs requested additional coaching support beyond the original project period. Given limited internal capacity and based on evaluation findings, we made significant changes to the structure of the second cohort as described in Practical Implications.

### 3.6 Data collection

While evaluation was conducted throughout the Collaborative to inform real-time improvements through an iterative process (e.g., post learning session feedback surveys), there were three primary timepoints for evaluation: baseline, mid-point, and end-line that included validated instruments used to capture organizational- and individual-level outcomes across Cohort 1.

Table 1 provides an overview of evaluation activities, participation, and response rates.

**Table 1:** Evaluation activities, participation and response rates (for evaluation results, see Supplemental materials).

Domain of measurement associated with R = MC*2 framework	Evaluation activity	Timepoint	Eligible participants	Responses^†^	Response rate
General capacity	Readiness Diagnostic Tool (RDT)	Baseline	16	9	56.25%
General capacity	RDT	Midline	13	5	38.46%
General capacity	RDT	Endline	13	8	61.54%
Policies, training and infrastructure	Trauma-informed Org. Assessment (TIOA)	Baseline	16	9	56.25%
Policies, training and infrastructure	TIOA	Midline	13	5	38.46%
Policies, training and infrastructure	TIOA	Endline	13	8	61.54%
Motivation	Professional Quality of Life Scale (ProQOL)	Baseline	16	9	56.25%
Motivation	ProQOL	Midline	13	5	38.46%
Motivation	ProQOL	Endline	13	8	61.54%
Motivation	Self-care assessment (SCA)	Baseline	16	9	56.25%
Motivation	SCA	Endline	13	8	61.54%
Innovation-specific capacity	Acceptability, Feasibility, and Appropriate-ness Scale (AFAS)	Endline	13	8	61.54%
Motivation	Focus Groups/Interviews	Baseline	16	6	37.50%
Motivation	Focus Groups/Interviews	Endline	13	3	23.08%

Readiness surveys were administered 7 months apart (Baseline; March 2024, Midline; October 2024, Endline; May 2025).

^†^Program level participation across learning collaborative activities is presented in Table 2.

### 3.7 Findings

Table 2 describes program participation across Learning Collaborative activities, including contextual information related to attrition. Due to variable participation across time points and small response counts at baseline (*n* = 9), midline (*n* = 5), and endline (*n* = 8), the analytic sample did not represent a stable cohort across assessments. Thus, we approach our findings as an exploratory implementation study, intended to identify directional patterns and contextual influences on implementation rather than to generate statistically generalizable conclusions. As a result, inferential statistical testing (e.g., repeated measures analyses or effect size estimation) was not feasible. Analyses therefore focused on descriptive trends across timepoints using aggregate comparisons.

**Table 2:** Program participation across Learning Collaborative activities (*n* = number of Collaborative participants).

Program ID	Baseline survey complete	Leaders included	Direct care included	Foundational learning	Mid-line survey	Coaching	Change initiative (program-wide)	Training of the trainers complete	Cont’d through endline	Endline survey completed
Program A: workforce capacity building; policy	Yes (*n* = 2)	Yes (*n* = 1)	Yes (*n* = 1)	Yes (*n* = 2)	No	No	No	No	No	No
	Program A Change Team consisted of two individuals; direct care staff participant was promoted to a senior position in another department following Foundational Learning sessions. The Program A leadership participant was unable to continue due to reduced capacity associated with increased responsibilities and personal health issues, and limited availability of other staff within the broader department to support.									
Program B: direct service; public awareness; policy advocacy	Yes (*n* = 1)	Yes (*n* = 2)	No	Yes (*n* = 2)	No	Yes (*n* = 2)	Yes	Yes (*n* = 1)	Yes (*n* = 2)	Yes (*n* = 2)
Program C: direct service; funding external partners	Yes (*n* = 1)	Yes (*n* = 1)	No	Yes (*n* = 1)	Yes (*n* = 1)	Yes (*n* = 1)	Yes	No	Yes	No
Program D: direct services	Yes (*n* = 2)	Yes (*n* = 1)	Yes (*n* = 1)	Yes (*n* = 2)	Yes (*n* = 1)	Yes (*n* = 2)	No	No	Yes (*n* = 2)	Yes (*n* = 2)
Program E: public awareness; policy advocacy; direct services	No	No	Yes (*n* = 1)	Some (*n* = 1)	No	No	No	No	No	No
	Program E leadership did not apply to participate. Program E direct care participant was accepted despite not meeting criteria due to indicating sincere interest as a professional development opportunity. Program E participant was ultimately unable to participate in the majority of the Collaborative activities due to workload and lack of staffing to provide coverage.									
Program F: direct service; public awareness; policy advocacy	Yes (*n* = 1)	Yes (*n* = 1)	Yes (*n* = 1)	Yes (*n* = 2)	Yes (*n* = 1)	Yes (*n* = 2)	Yes	Yes (*n* = 2)	Yes (*n* = 2)	Yes (*n* = 2)
Program G: direct services	Yes (*n* = 2)	Yes (*n* = 1)	Yes (*n* = 4)	Yes (*n* = 5)	Yes (*n* = 2)	Yes (*n* = 5)	Yes	Yes (*n* = 2)	Yes (*n* = 5)	Yes (*n* = 2)

Blue = intervention-related; green = evaluation; grey = context.

For programs that identified a change initiative and continued through endline, Table 3 provides a summary of specific needs, strategies, and preliminary outcomes.

**Table 3:** Summary of program needs, strategies, and preliminary outcomes.

Program type & need	Strategy/adaptation	Preliminary outcomes
Program B: direct service; public awareness; policy advocacy Need: Culture of equity and inclusion within program. Identified by all-staff survey and committee input.	Integration of trauma-informed and equitable approaches into all-staff survey design and launch Integration of TIE approaches into committee charged with equity and inclusion efforts	Staff survey data utilized to inform strategic plan priority activities for department Launch of tailored training sessions for staff and leadership based on findings
Program C: direct service; coordination / funding of external partner direct services Need: Consistent implementation of TIE approaches among subcontracted partners. Identified by program data scan (client feedback, case notes, service outcomes).	Integration into partner contracts of clarified language regarding expectations for TIE care Implementation of TIE training for all subcontracted partners Preliminary design of client feedback survey and engagement with partners to standardize ongoing client feedback mechanism	Increased knowledge and skills among subcontracted partners of TIE approaches assessed via surveys and ongoing program monitoring Given clarified contract expectations, increased pathways for addressing quality of care concerns
Program F: direct service; public awareness; policy advocacy Need: Fostering a program culture of care, safety, healing, and equity. Identified by all-staff survey.	Launch of TIE working group focusing on workplace culture and morale Engagement with all staff to inform goals and priorities Implementation of learning series for staff and leadership Integration of TIE practices into workplace meetings	Ongoing team support spaces and activities, assessed via surveys, program reports and group and 1:1 staff feedback Inclusion of TIE principles in program’s overarching strategic plan
Program G: direct service Need: Consistent response to support staff and constituents following traumatic incidents. Identified by staff and leadership input, policy and protocol review.	Engaged staff and leadership in identifying types of critical incidents and categorizing by tiers 1, 2, and 3 for differentiating responses Design of tiered protocol response based on best practices and program context Creation of companion materials and guidance for implementation	Implementation of Critical Incident Protocol (CIP) across all program sites Majority of sites report awareness of CIP and utilization following recent incidents

Table adapted from Harris et al. (2025). Implementing the Modular Approach to Therapy for Children (MATCH-ADTC) within a Learning Collaborative retrieved March, 16, 2026 from, https://www.bakercenter.org/programs/policy-makers/quality-care-initiative/case-studies.

Below is a synthesis of the findings from the assessments, feedback surveys, and focus groups, with more detail available in the full Baker Center Cohort 1 report provided in Supplementary materials.

Across multiple assessments, some progress was observed at both organizational and participant levels. Measures of readiness and capacity indicated moderate improvements in organizational functioning, particularly in culture, climate, structure, and cross-site relationships, alongside stable leadership support (Readiness Diagnostic Tool (60)). The trauma-informed organizational assessment showed gains in policies, procedures, supervision, and training (Trauma-Informed Organizational Assessment (63)). Participant-facing measures aligned with these findings: high acceptability and appropriateness scores indicated alignment with staff values and program goals [AFAS (64)], while learning sessions, coaching, and training-of-the-trainer activities improved knowledge, confidence, and practical skill application. Participants reported increased understanding of trauma-informed and equitable principles, stress impacts, and self-care [SCA (65, 66)].

Qualitative findings contextualized these outcomes, highlighting the importance of interactive facilitation, practical tools, and peer exchange. Direct care staff emphasized that protected space to share challenges and learn from other sites enhanced relevance and application. As one participant noted, “they encouraged participation and made me feel comfortable to share and engage” (focus group staff participant). Peer learning and cross-site collaboration reinforced confidence and fostered shared commitment to trauma-informed, equity-centered practices.

Despite these strengths, the results also illustrated persistent challenges. Staff-level capacity declined by endline, suggesting a disconnect between organizational improvements and frontline experience (60). Participants emphasized the need for greater support for direct care staff, with one supervisor noting that “there’s so much more advocacy that we have to do [for direct care staff]” (focus group leadership participant). Some staff often felt primarily responsible for implementation without adequate organizational backing and noted structural barriers constraining sustainable implementation, such as heavy workloads and shifting organizational priorities. Quantitative findings reinforced these concerns, including declines in organizational commitment (63), fluctuating resource utilization (60), and persistently low compassion satisfaction with high burnout (67, 68).

## 4 Methodological constraints

As illustrated in Tables 1, 2, the analytic sample was small and did not represent a stable cohort across assessments, limiting inferential statistical testing. This was in part due to participant availability and attrition. Typical strategies to increase response rates such as incentives were unavailable due to restrictions on use of Federal funding for City employees. Due to funding constraints, the implementation timeline and evaluation timeline were developed asynchronously, meaning missed opportunities to embed evaluation activities such as focus groups during times of greater participant availability.

## 5 Practical implications and lessons learned for future applications

Based on these findings, we offer two key implications and lessons learned that may be relevant for other practitioners seeking to apply quality improvement models within their own settings.

### 5.1 Importance of maintaining a multi-level focus: supporting direct care staff and immediate needs while also promoting long-term systems change

Our interpretation of the mixed findings is that while interventions such as the Collaborative can be an important entry point for introducing trauma-informed and equitable principles and approaches, it is critical to continuously balance time and resources across the multiple levels of influence. Specifically, to balance providing immediate support and learning to participants alongside advocacy for long-term systems changes.

Implementation studies emphasize that trauma-informed care is most effective when supported by broader organizational changes including leadership engagement, workforce development strategies, and institutional policy reforms (69, 70). However, for many City workers, pay, benefits, hours, and other structural factors influencing workforce well-being are determined by their Union’s respective collective bargaining agreements. The City of Boston’s Office of Labor Relations negotiates and administers the City’s collective bargaining agreements with the Unions that represent City of Boston employees. For non-unionized workforces, salary is determined by a variety of factors, including funding availability and requirements, parity with salaries set by collective bargaining agreements, and human resources assessments.

As a fellow City program but not part of Labor Relations, Union leadership, or Human Resources, engaging in salary-related negotiations or advocacy was outside the scope of our work. However, as is common in resource-constrained, real-world settings, we adapted a pragmatic approach, prioritizing action and responding to the needs of Collaborative participants within our locus of control and influence.

Reflecting on the outcomes, a lesson learned would be to more clearly define “leadership” and more consistently engage Collaborative leadership in advocating for long-term systems-level changes. While executive-level leadership was notified of the Collaborative, the leadership who ultimately participated were program-level leadership still operating within the broader hierarchy that limits their authority to make structural changes. Notably, the program with the most far-reaching and arguably impactful systems-level intervention (a Critical Incident Protocol implemented across all sites) was also the program that involved both mid-level leadership and executive leadership in the Collaborative process.

Another lesson learned is to more intentionally address throughout the initiative the disproportionate burden of change activities on direct care staff, who are also supporting constituents with whom they may share similar experiences and stressors. While evaluation findings indicate that the intervention provided some useful tools and resources for staff and there was a moderate increase in self-care practices, there remained persistent burnout and compassion fatigue. Thus, a companion strategy to the long-term systems advocacy described above might be including guidance during coaching sessions on developing intentional supports for direct care staff during the change process, including addressing workload concerns and ensuring they have a meaningful voice in decision-making.

### 5.2 Ensure internal implementation team capacity, particularly for coaching

The evaluation found participants especially valued coaching given it focused on tailored operationalization of learning content and organizational changes. However, coaching with multiple sites requires significant time and resources for coordination, preparation, facilitation and follow up. Federal funding was only sufficient for one full-time employee and consultants, and is time-limited, thus presenting a serious challenge for sustainability of this crucial element of the initiative. Moving forward, we are both modifying the scope for cohort 2 to be more targeted to coaching, providing more role-specific sessions, and identifying a diversified funding stream to sustain adequate personnel.

## Data Availability

The original contributions presented in the study are included in the article/Supplementary material, further inquiries can be directed to the corresponding author.
