# Supplementary material for: Building trauma-informed and equitable capacity for city programs: enhancing workforce well-being and service quality in Boston
Source: Front Public Health. 2026 May 29;14:1777682. doi: 10.3389/fpubh.2026.1777682 (PMC13261807; doi:10.3389/fpubh.2026.1777682)
Supplement: Supplementary file 1 [file Supplementary_file_1.pdf]

THE BAKER CENTER
FOR CHILDREN AND FAMILIES

Improving care. Changing lives.

Boston Public Health Commission's Trauma Informed and Equitable Learning Collaborative

COHORT 1 EVALUATION REPORT

September 2025

PREPARED FOR:
Krupa Boradia, Bronwen White, and Kimberly Mendoza-Iraheta
BPHC TIE Learning Collaborative Leadership

PLEASE DIRECT ALL CORRESPONDENCE REGARDING THIS SUMMARY TO:

Michaela Harris, MPH.
Senior Implementation and Quality Improvement Specialist
mharris@bakercenter.org

Sean Snyder, DPA, MSW, LCSW
Director of Implementation and Technical Assistance
ssnyder@bakercenter.org

53 Parker Hill Avenue,
Boston, MA 02120

info@bakercenter.org
617-232-8390
bakercenter.org

Executive Summary 3
Acronyms Table 5
Introduction 6
Purpose 6
Methods 6
Purpose of Quantitative Methods 7
Purpose of Qualitative Methods 8
Guiding Framework: Practical, Robust Implementation Sustainability Model (PRISM) and Reach, Effectiveness, Adoption, Implementation, and Maintenance (RE-AIM) 9
Key Findings 11
Reach & Adoption 13
Learning Session Results 14
Coaching Session Results 17
Training of the Trainer (ToT) Results 19
Effectiveness & Implementation 20
Organizational Level Outcomes 20
Individual Level Outcomes 22
Endline Focus Group Findings 25
Maintenance 27
Key Recommendations: 28
Appendix 29
Learning Session Training Feedback 29
Appendix Table 1. Coaching Takeaways 30
Cross Site Readiness Report 31
Appendix Table 2. Change Over Time in Participant Perspectives by Role (2024–2025) ... 40
Reference List 41

Executive Summary

BPHC Trauma Informed and Equitable (TIE) Learning Collaborative – Cohort 1 Evaluation Report

The TIE Learning Collaborative aimed to support Boston city workers in acknowledging workplace exposure to trauma and conflict, while equipping them with strategies and tools for self-care, equity, and sustainable trauma-informed practices. Core objectives included providing access to mental health resources, building practical coping strategies, offering tailored coaching, and cultivating healthier organizational cultures.

Purpose & Methods

This report synthesizes Cohort 1 (March 2024 – August 2025) evaluation data to answer two central questions:

- Was Cohort 1 implemented as intended? (Process)
- Were intended outcomes achieved? (Outcome)

A mixed-methods design, quantitative was collected via surveys (Readiness Diagnostic Tool[1], Trauma Informed Care Organizational Self-Assessment[2], Professional Quality of Life (ProQOL)[3], Self-Care Assessment[4], Acceptability Feasibility Appropriateness Scale (AFAS)[5]) and qualitative was collected via focus groups and interviews. Data were analyzed using SPSS and rapid qualitative methods. Response rates averaged ~54%, limiting statistical power but yielding valuable directional insights.

Key Findings

- **Reach & Adoption**: Seven city agencies participated in Learning Sessions. Senior leaders were highly represented, direct-care staff underrepresented. Attendance was strongest in early sessions, declining over time. Coaching engagement was uneven, with BCYF and MOIA most consistent.
- **Effectiveness & Implementation**: Participants reported gaining "a good bit" of knowledge, with coaching and peer-to-peer discussions cited as most impactful. Confidence in applying TIE skills was highest among direct-care staff engaged in coaching.

---

[1] Scaccia, J. P., Cook, B. S., Lamont, A., Wandersman, A., Castellow, J., Katz, J., & Beidas, R. S. (2015). A practical implementation science heuristic for organizational readiness: R = MC2. Journal of community psychology, 43(4), 484-501.

[2] Cellarius, K., Davis, M., Tuttle, A., daRosa, C. (2023) PSU/TIO Trauma Informed Care Implementation Assessment Instrument, v1.2. Portland, OR: Portland State University.

[3] Stamm, B. H. (1995). Professional Quality of Life Scale (PROQOL) and Galiana, L., Oliver, A., Arena, F., De Simone, G., Tomás, J. M., Vidal-Blanco, G., Muñoz-Martínez, I., & Sansó, N. (2020). Development and validation of the Short Professional Quality of Life Scale based on versions IV and V of the Professional Quality of Life Scale. Health and quality of life outcomes, 18(1), 364. https://doi.org/10.1186/s12955-020-01618-3

[4] Adapted from Saakvitne, Pearlman, & Staff of TSI/CAAP (1996). Transforming the pain: A workbook on vicarious traumatization. Norton.

[5] Cho, E., Lyon, A. R., Tugendrajch, S. K., Marriott, B. R., & Hawley, K. M. (2022). Assessing provider perceptions of training: Initial evaluation of the Acceptability, Feasibility, and Appropriateness Scale. Implementation Research and Practice, 3, Article 26334895221086269. https://doi.org/10.1177/26334895221086269

- **Training of Trainers (ToT)**: 15 participants (predominantly direct-care staff) demonstrated significant confidence in applying and training others on trauma-informed practices, showing scalability [6].
- **Organizational Outcomes**: Capacity and infrastructure improved moderately, particularly in supervision, training, and policy integration. Leadership commitment declined and staff capacity fell sharply by endline, revealing a gap between system-level progress and frontline support.
- **Individual Outcomes**: Self-care practices improved modestly, but burnout and compassion fatigue remained high. Compassion satisfaction was consistently low.
- **Perceptions of Applicability (AFAS)**: High acceptability, feasibility, and appropriateness confirmed strong alignment with agency missions and participant needs, even at endline[5].

Sustainability & Limitations

While the program's design and peer-learning opportunities were highly valued, sustainability challenges included existing workloads, leadership turnover, limited direct-care representation, and uneven exposure to the full curriculum. Findings are directional, not statistically generalizable, due to small sample size and self-selection bias.

Key Recommendations

- **Increase representation across roles**, especially direct-care staff - and create role-specific learning spaces.
- Standardize content delivery to ensure **consistent exposure across agencies**.
- **Clarify ToT roles and expectations** to strengthen internal training capacity.
- **Improve evaluation systems** by embedding real-time feedback, setting minimum participation thresholds, incentivizing survey completion, and collecting expanded demographics.

[6] Betz, M. G (2025). City Learning Collaborative: Promoting Trauma-Informed & Equitable Approaches and Workforce Well-Being 2025.Capacity Building & Training Initiative. Self- Published.

Acronyms Table

Acronym	Full Term	Notes/Context
BPHC	Boston Public Health Commission	Host agency for the Learning Collaborative
TIE	Trauma Informed and Equitable	Name of the Learning Collaborative
LC	Learning Collaborative	Refers to the initiative model/activities
RDT	Readiness Diagnostic Tool	Assesses organizational readiness and capacity
TIC	Trauma-Informed Care	Organizational framework/practice
TIOA	Trauma Informed Organizational Assessment	Evaluates TIC elements within agencies
R&C	Readiness and Capacity	Shorthand for RDT-based assessment
ProQOL	Professional Quality of Life	Measures compassion satisfaction, burnout, compassion fatigue
SCA	Self-Care Assessment	Evaluates individual self-care practices
AFAS	Acceptability, Feasibility, and Appropriateness Scale	Measures perceptions of training utility/relevance
ToT	Training of the Trainer	Role for internal capacity-building
PRISM	Practical, Robust Implementation and Sustainability Model	Framework guiding analysis
RE-AIM	Reach, Effectiveness, Adoption, Implementation, and Maintenance	Framework guiding analysis
DEI / DEIB	Diversity, Equity, Inclusion (and Belonging)	Referenced in coaching/committee context
EAP	Employee Assistance Program	A resource for workplace conflict resolution available to Boston City workers

Introduction

The Trauma Informed and Equitable Learning Collaborative (TIE) has clearly defined objectives outlined in the developed logic model and training materials. The primary focus of this Learning Collaborative is a) to acknowledge workplace exposure to trauma and conflict of Boston city workers and b) provide training participants with the knowledge, support, and skills to continue their work in a safe and equitable environment.

The primary objectives of the Learning Collaborative are the following:

- To provide Boston city workers **access to relevant mental health resources** for self and collective-care, collaboration, and support within the Greater Boston community.
- To gain practical tools and **strategies for coping, wellbeing, and trauma response**.
- To receive tailored coaching to **promote and apply trauma-informed and equitable policies, programming, and practices**.
- To provide resources that **cultivate a healthier organizational culture and climate**.

Purpose

The purpose of this Cohort 1 report is to collate data across learning collaborative activities and interpret results in order to provide the project team with key insights and recommendations to integrate into Cohort 2 planning and beyond.

This report is guided by two central evaluation questions that were identified collaboratively by the project team to reflect both implementation fidelity and program outcomes:

- **Was Cohort 1 implemented as intended?**
  - Examines whether the learning collaborative was delivered the way it was designed (e.g., frequency and format of sessions, level of engagement from sites, use of resources). In practical terms, it asks whether participants experienced Cohort 1 as it was meant to be delivered.
- **Were Cohort 1 intended outcomes achieved?**
  - Examines whether the collaborative led to desired changes (e.g., increase in TIC knowledge and skills, coping skills, and progress towards organizational goals). Measures were drawn from collected participation data, feedback surveys, and focus groups to determine whether these intended impacts occurred.

Methods

Throughout Cohort 1 of the TIE Learning Collaborative, a mixed methods approach was integrated into evaluation activities. Quantitative data was collected via surveys and analyzed using SPSS statistical software, while qualitative data was collected via interviews and analyzed using Dedoose software with rapid qualitative analysis[7].

---

[7] Kowalski, C. P., Nevedal, A. L., Finley, E. P., Young, J. P., Lewinski, A. A., Midboe, A. M., & Hamilton, A. B. (2024). Planning for and Assessing Rigor in Rapid Qualitative Analysis (PARRQA): a consensus-based framework for designing, conducting, and reporting. Implementation science : IS, 19(1), 71. https://doi.org/10.1186/s13012-024-01397-1

Purpose of Quantitative Methods

Quantitative methods, via surveys, were used to assess three key areas:

- Organizational readiness and capacity to implement new interventions
- Staff knowledge and skills related to collective/self-care, and
- Professional quality of life (compassion fatigue, burnout, and compassion satisfaction).

Data Collection

Surveys with validated instruments were used to capture organizational- and individual-level outcomes across Cohort 1, with details available in the Evaluation Plan (with the exception of the AFAS, which was added at endline). These included the following:

- **Readiness Diagnostic Tool (RDT)**[1] to assess organizational capacity (*validated*)
- **Trauma-Informed Organizational Assessment (TIOA)**[2] to examine policies, training, and infrastructure (*practice-based tool, not yet validated*)
- **Professional Quality of Life Scale (ProQOL)**[3] to measure compassion satisfaction, burnout, and fatigue (*validated*), and
- **Self-Care Assessment (SCA)**[4] to evaluate staff self-care practices across multiple domains (*validated*).
- At endline, the **Acceptability, Feasibility, and Appropriateness Scale (AFAS)**[5] was also administered to assess staff perceptions of training relevance and utility (*validated*).

Together, these instruments provided a comprehensive picture of readiness, organizational infrastructure, staff wellbeing, and training utility.

Baseline and Endline assessments focused closely on organizational readiness and capacity to implement TIE approaches, professional quality of life, self-care practices. The Endline assessment was modified to include the AFAS scale in order to determine perspectives of utility of program learning into current work and practice. Midline assessments focused on organizational readiness and capacity, current use of coping strategies, and implementation of TIE practices post training. Response rates for the following are shown below:

R&C Assessment	Respondents (N = 24)
Baseline	9 respondents
Midline	5 respondents
Endline	8 respondents

Quantitative Analysis

- **Readiness Surveys** were administered 7 months apart (Baseline; March 2024, Midline; October 2024, Endline; May 2025).
- Analysis was conducted by the evaluation team using **SPSS software**, mean distributions were calculated for **each instrument and subscale**.
- Aggregate comparisons were examined using **one-way descriptive analyses**.

Purpose of Qualitative Methods

Qualitative methods were used to understand staff experiences in the TIE Learning Collaborative, including:

- Learning Collaborative experiences,
- Implementation of TIE skills in service settings,
- Organizational barriers and facilitators to implementation, and
- Resource utilization and sustainability.

Baseline (June – August 2024)	
**Role**	**Participants (N = 6)**
Direct-care Staff	1
Supervisors/ Direct-care Staff	1
Senior Leader	4

Data Collection

- **Focus Groups** (60 minutes)
- **Key Informant Interviews (KIIs)** (~45 minutes) with participants grouped by role (direct care staff, supervisors, senior leadership) and training role (Training of Trainers, ToT).
- Both methods were conducted twice: **Baseline (June–August 2024)** and **Endline (June–August 2025)**.

Endline (June – August 2025)	
**Role**	**Participants (N = 2)**
Direct-care Staff	1
Training of the Trainer (ToT)	1
Senior Leader	1

Qualitative Analysis

- Sessions were conducted and recorded in Zoom, transcribed by **Rev.com**, and verified by the Baker Center evaluation team.
- Coding and analysis were conducted by the evaluation team using **Dedoose** software, supplemented by **Rapid Qualitative Analysis (RQA[7])** for timely interpretation of high-level themes.
- Recruitment was led by **BPHC program leadership**.

Guiding Framework: Practical, Robust Implementation Sustainability Model (PRISM) and Reach, Effectiveness, Adoption, Implementation, and Maintenance (RE-AIM)

The PRISM and RE-AIM frameworks will be used to organize and discuss how agency contextual factors interact to develop eventual Cohort 1 outcomes. **RE-AIM** was the original framework developed to evaluate public health and implementation outcomes. **PRISM** was later built upon RE-AIM, extending it by incorporating organizational and contextual factors that influence implementation and sustainability. The following figure shows how these frameworks intersect to guide analysis:

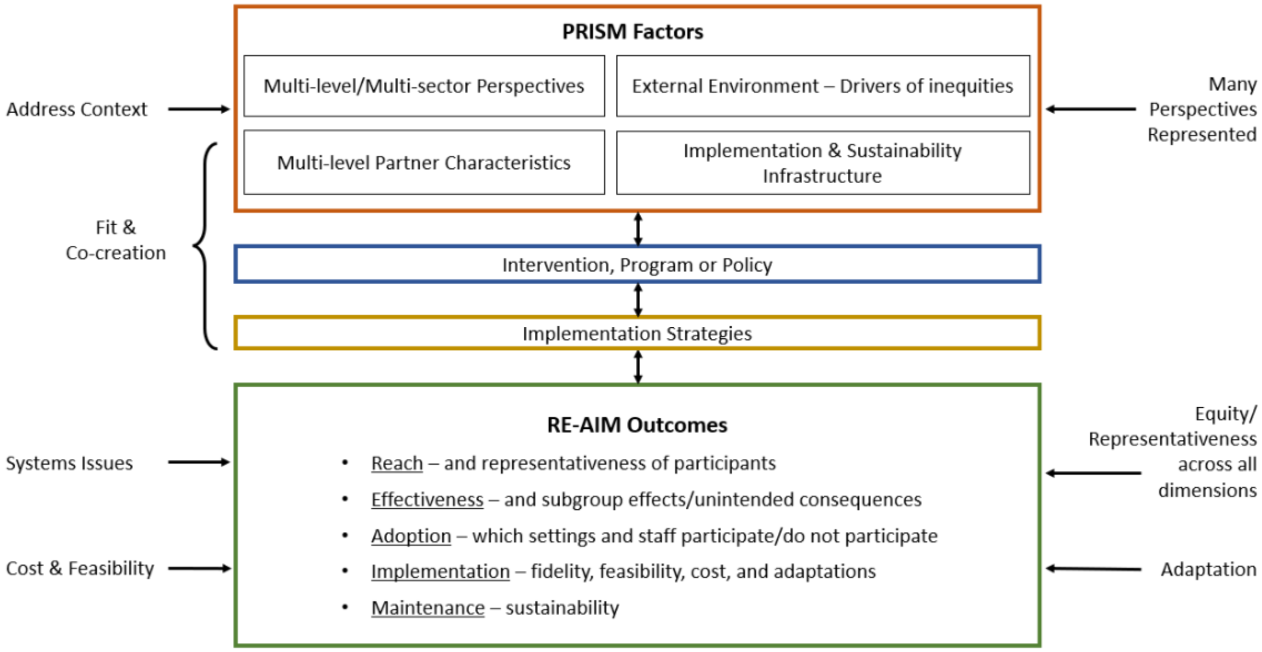

Figure 1. RE-AIM and PRISM Explained[8]

Addressing Context. Baseline assessments and Baker Center-led interviews / focus groups were used to better understand organizational culture and climate at the beginning (and throughout) BPHC-led learning session activities. PRISM factors will distill these findings in Table 1. (Pages 10-11).

Addressing Fit. Feedback on BPHC-led coaching and ToT sessions were used to assess the impact of program learning on participants, and their applications to current work and change initiative(s).

Addressing Outcomes. Findings from collected attendance records, program assessments, and Baker Center-led focus groups were used to better understand the following RE-AIM Domains[9,10].

- **Reach and Adoption**: Assessing the extent to which the LC engaged a diverse mix of individuals and organizations. Here, it reflects which agencies and role types (senior leaders, supervisors, direct-care staff, ToTs) participated and how broadly sites initiated and took up the LC content by endline.

---

[8] RE-AIM. (n.d.). What is PRISM? https://re-aim.org/learn/prism/

[9] Glasgow, R.E., Trinkley, K.E., Ford, B. et al. The Application and Evolution of the Practical, Robust Implementation and Sustainability Model (PRISM): History and Innovations. Glob Implement Res Appl 4, 404–420 (2024). https://doi.org/10.1007/s43477-024-00134-6

[10] Rabin, B.A., Cakici, J., Golden, C.A. et al. A citation analysis and scoping systematic review of the operationalization of the Practical, Robust Implementation and Sustainability Model (PRISM). Implementation Sci 17, 62 (2022). https://doi.org/10.1186/s13012-022-01234-3

- **Effectiveness & Implementation** Examines both the impact of the LC and the consistency of its delivery. In this report, it captures shifts in organizational and individual outcomes (R&C, ProQOL, Self-Care, AFAS) as well as how participants experienced content, coaching, and application.
- **Maintenance**: Focuses on the extent to which changes can be sustained over time. Here, it is expressed as “Key Recommendations” that translate Cohort 1 findings into guidance for Cohort 2.

Key Findings

Setting the Stage with PRISM Factors

Table 1. PRISM/Contextual Factors of Learning Collaborative Participants at Baseline.

PRISM Category	Quantitative Findings (Baseline Readiness Surveys)	Qualitative Findings (Baseline Focus Groups)
**Cross-Site, Multi-level Organizational Perspectives**	• Leadership identified as a strength across sites.<br>• Perceptions of organizational climate & structure moderate, suggesting inconsistency in understanding "how things are done".<br>• Conflict resolution strategies rely heavily on internal discussions and/or externally led facilitation.	• Staff view senior leadership as holding power for change but often disconnected from frontline realities.<br>• Supervisors positioned as key intermediaries between staff, community, and leadership.<br>• Direct care staff reported feeling overlooked in decisions, especially those impacting communities of color and immigrant groups.
**Cross-Site, Multi-level Organizational Characteristics**	• Self-care assessment suggests that across sites, there is an "occasionally" sense of balance between personal and professional needs. Physical and Workplace self-care were most reported.<br>• Staff burnout risk with opportunity to identify root cause and needed coping skills.	To create a trauma informed workplace environment, focus group participants shared that there needs to be:<br>• Knowledge of the impact of trauma at all levels (community, organizational, individual),<br>• Sensitivity to the context of the community served, and<br>• Knowledge of how trauma could impact workplace culture and climate.
**External Environment - Drivers of Change**	• TIC community collaboration scored low, providing an opportunity through the Learning Collaborative to share resources and perspectives across sites.<br>• Awareness of Trauma Specific Services scored low, providing opportunity to share tailored resources available in the city of Boston.	• Staff at all levels expressed concern for current conflict resolution policies imposed by the city government (e.g., EAP).<br>• Leadership concerned about staff retention challenges.

**Implementation & Sustainability Infrastructure**	• Resource utilization moderately strong, suggesting there is visibility of how organizational resources will be used to support the change initiatives.<br>• Commitment to DEI and TIC policies present, but integration into training/evaluation practices are incomplete.<br>• Performance reviews scored low, creating opportunity to cultivate feedback mechanisms.<br>• Processes in place (policy review, TIC training, evaluation), but possibly not sustained.	• Participants expressed the need for applied coaching to ensure skills "stick".<br>• Participants looking for support in creating critical incident protocols, giving feedback using a trauma informed lens, understanding traumatic stress in the workplace, and navigating power dynamics.

<u>**In summary**</u>: At baseline, sites **reported strong leadership buy-in and organizational capacity** to participate in learning collaborative activities, despite varied infrastructure across sites (N = 7). While DEI and trauma-informed policies were present, scores suggest that they were **not consistently embedded into supervision or evaluation practices**. Direct-care staff, especially staff of color and/or immigrant background, reported a disproportionate need to be on the "front lines" for community members and their needs yet described feeling "disconnected" from organizational decision-making.

At baseline, participants **consistently expressed a need for practical, hands-on tools** (e.g., critical incident protocols, conflict resolution strategies) and stronger support for staff wellness. These findings suggested that a targeted investment in staff support, equity centered practices and applied implementation coaching would be **critical to ensuring sustainability and long-term impact of learning collaborative skills and change initiatives.**

Reach & Adoption

In reviewing the following domain, please consider the following guiding questions:

- Did Cohort 1 reach the right mix of roles (leaders, direct-care staff, ToTs) across activities, and how can representation be strengthened in Cohort 2?
- How well do participating sites reflect the broader system you want to impact in Boston?
- What strategies could encourage greater engagement from direct care staff, who were under-represented in learning and coaching sessions?

Role Types:

- **Senior Leaders**: Defined as participants who understand the greater organizational context and what "levers" need to be pulled to make change happen.
- **Direct-care Staff**: Defined as participants who directly interact with children and families (i.e., constituents) in the greater Boston community and understand how ideas can be put into practice. This also includes Direct-care staff that supervise other Direct-care staff.
- **Training of the Trainers (ToT)**: Defined as participants (senior leader, supervisor, or direct-care staff), who participated in Training of the Trainer (ToT) sessions and who can provide internal TIE training to their organization. This role was <u>not</u> required to attend foundational learning sessions in Cohort 1.

Key Learning Collaborative Activities:

- Learning Sessions
- Coaching Sessions
- Training of the Trainer (ToT) Sessions
- Agency Change Initiatives (if applicable)

Learning Session Results

Learning sessions (LS) were administered by BPHC staff in-person and virtually across 4 months from March to June 2024. The purpose of Learning sessions was to give participants foundational knowledge and skills in trauma-informed care and equity centered principles.

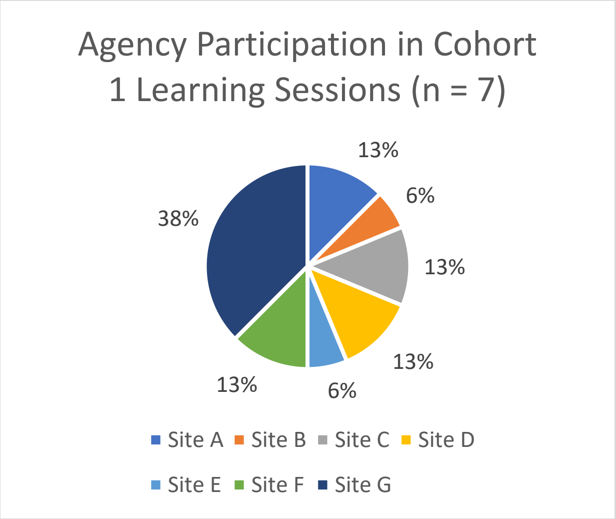

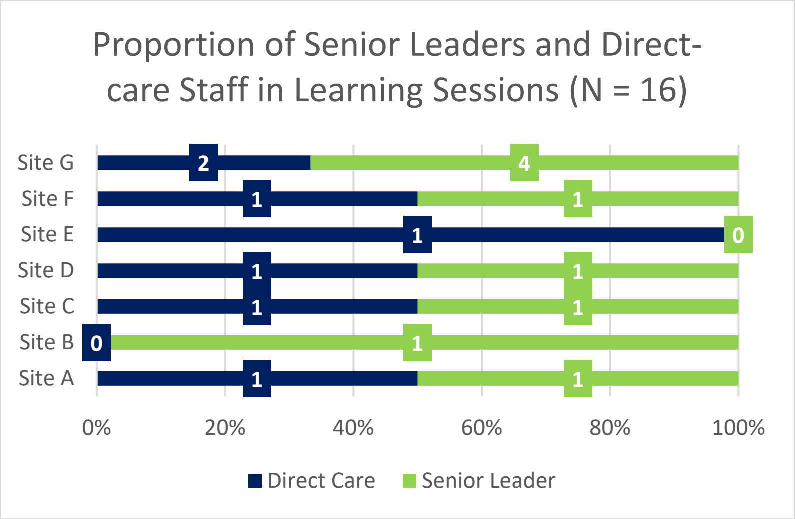

Figure 1. Learning Session Participation by Agency and Role Type.

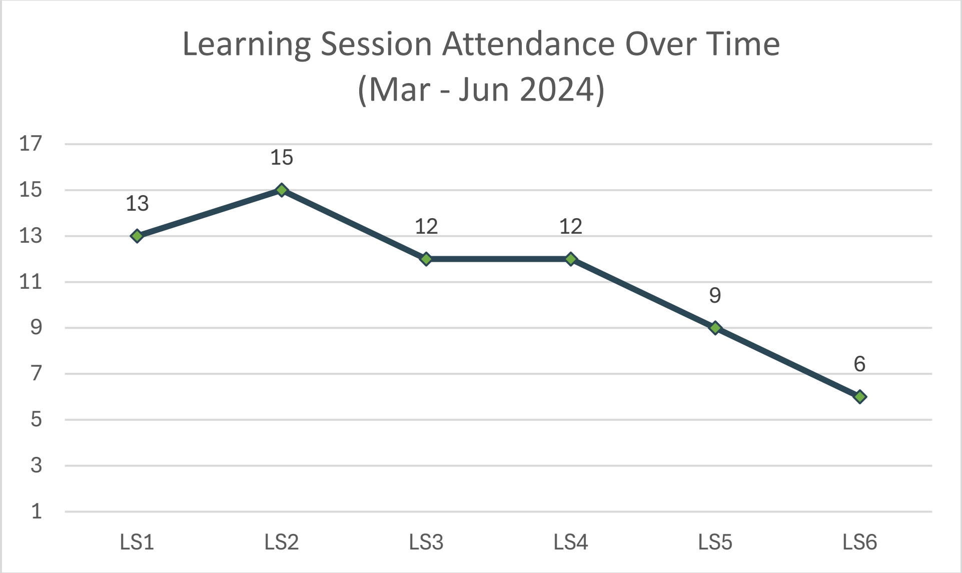

Figure 2. Learning Session Attendance Over Time

Learning Session 2 (LS2) received the most attendance at 15 participants, while the Learning Session 6 (LS6) – "Intro to Coaching" received the least amount of attendees at 6 participants. Following each Learning Session, participants were asked to complete a training evaluation survey that asked them to list their knowledge of training objectives before and after training. Results from this assessment for each learning session can be found in the Appendix of this report.

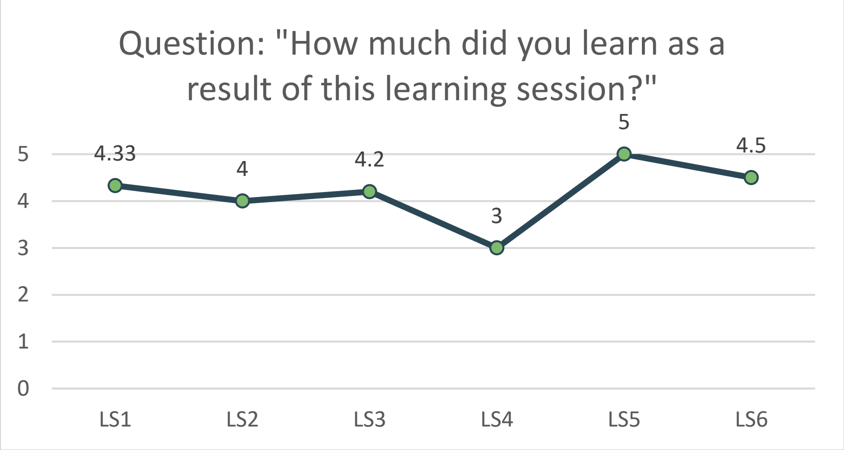

Scale				
Very Little	Little	Some	A good bit	A great deal
1	2	3	4	5

Figure 3. “How much did you learn as a result of this learning session?”

Following each Learning Session, participants were asked to complete a training evaluation survey which asked participants to share their knowledge of trauma-focused skills before and after training. Results from this assessment for each learning session can be found in the Appendix of this report. On average, participants who completed the post training surveys generally **left sessions feeling like the gained “a good bit” of learning**.

KEY TAKEAWAYS FROM FOCUS GROUPS – LEARNING COLLABORATIVE<br>JULY 2024
Participants across groups shared that **content covered in learning sessions endorsed the knowledge and skills they knew**. However, participants are looking forward to coaching sessions and the opportunity to hear from other sites their experiences and how the skills apply to different settings.<br><br>*"I could say that probably this part is the most helpful for me. It was nice, all of the materials, but I had hoped for a space where we were actually going to talk about how that relates to our jobs and have more discussion around the challenges we were having. (..) that's what I was waiting for, so I'm happy we're in that space now, so thank you."*<br>*– Agency Senior Leader*
Participants endorsed the following **topics that could be useful in coaching sessions**:<br>• Tips for giving feedback using a trauma informed lens (from supervisor to supervisee)<br>• Creating a critical incident protocol<br>• Understanding trauma and traumatic stress in the workplace (e.g., supporting colleagues and understanding signs of traumatic stress)<br>• Navigating power dynamics within an organization (bottom up vs. top-down information sharing)<br>• Conflict resolution strategies (before escalating to EAP)
Participants shared the desire for **more in-person learning sessions in the future** to encourage engagement and more time for discussion across sites to share best practice.

Specific Highlights:

- Across the 6 Learning Sessions, **average attendance was ~75%** when excluding LS6/Intro to Coaching.
- **Engagement was strongest for LS1 and LS2 (88-93% attendance)**, then gradually declined to under 50% by LS4. Suggesting that agencies were eager to begin Learning Collaborative activities but competing priorities or staff fatigue may have reduced sustained attendance.
- Consistently Engaged Sites:
    - **Site F attended 92% of offered sessions.**
    - **Site C attended 75% of offered sessions**.
    - **Site G has the largest number of participants from a single organization** (6) and attended 72% of offered sessions.
- Lowest Engaged Sites:
    - **Sites I & J** attended ~50% of offered sessions on average, with some sessions unattended.
- In summary, "dose" or exposure to the full TIE curriculum was uneven across sites in Cohort 1 and weighted heavily towards organizational leadership participation. However, across learning activities, participants left feeling like they **gained "a good bit" of learning**.

Coaching Session Results

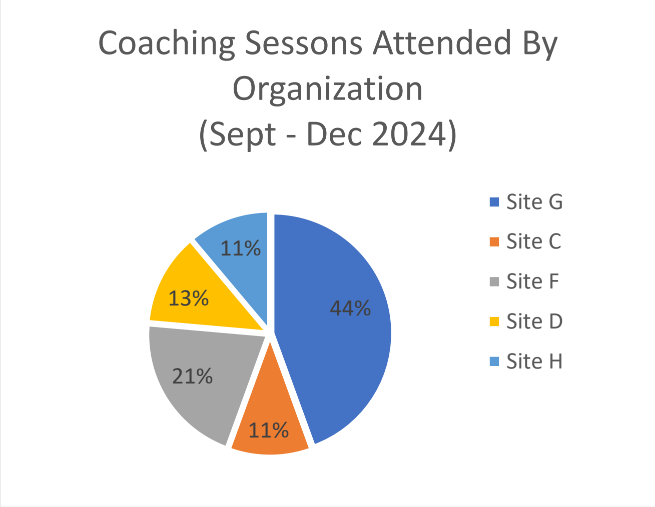

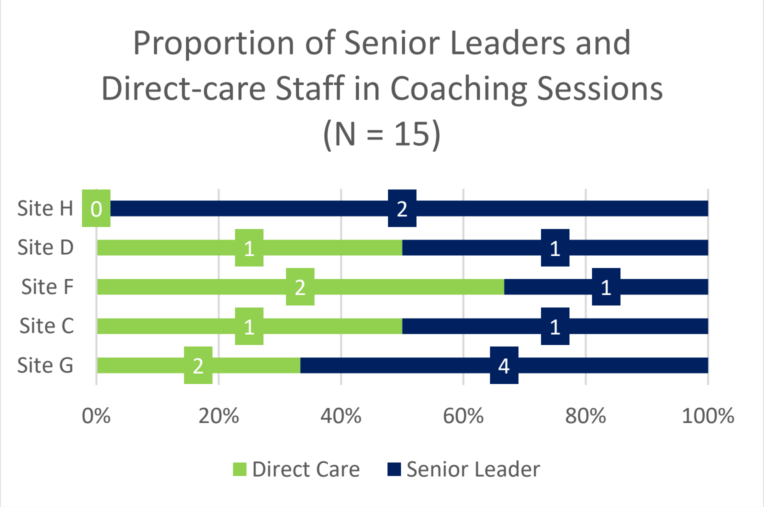

Figure 4. Coaching Session Participation by Agency and Role Type.

Coaching Sessions were administered in-person and virtually across **4 months from September to December 2024 across 3 different formats: individual coaching, cross site coaching, and leadership coaching**. Most notably, Site G attended the most available coaching sessions (N = 9) followed by the Site F (N = 8).

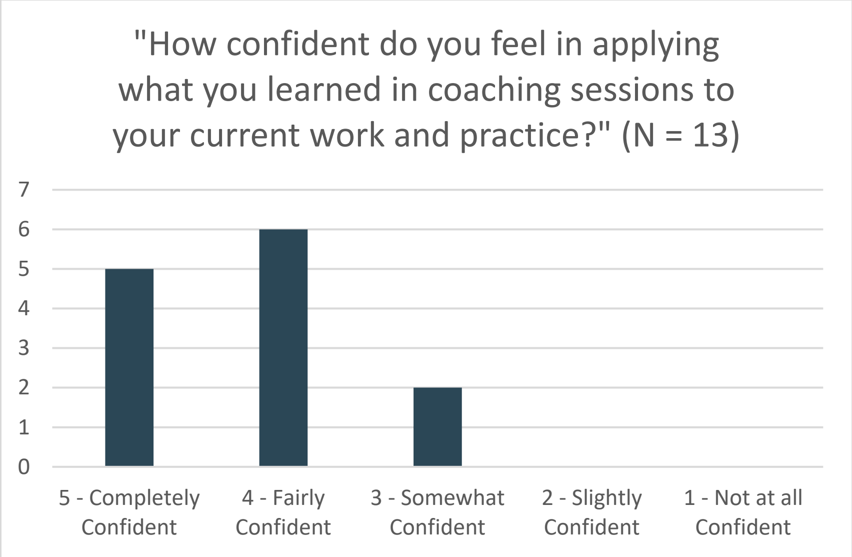

Figure 5. "How confident do you feel in applying what you learned in coaching sessions?"

Following the Coaching Session period, participants were asked to complete a coaching evaluation survey that asked about their experience participating in Coaching sessions and key learnings. Results from this assessment can be found in the Appendix Table 1 of this report. Respondents were also asked to answer the question "*How confident do you feel in applying what you learned in coaching sessions to your current work and practice?*" where they answered from 1 – "Not at all confident" to 5 – "Completely confident". Those

responses can be found in Figure 5 above. On average, **84% of survey respondents felt "fairly" or "completely" confident in what they learned.**

KEY TAKEAWAYS FROM COACHING FEEDBACK SURVEY (FEBRUARY – JUNE 2025)
Adoption of TIE Skills:<br>• Direct care staff respondents (N = 3), although representing lower participation in coaching sessions (N = 5), **were more likely to report higher confidence in adopting TIE skills (3 or more skills; 100%) by the end of coaching sessions.** Senior leader respondents (N = 9) displayed a more moderate perspective of skill uptake (2, 3, and 4+ skills; 33% each).<br>• Agencies with greater role diversity (i.e., Site D & Site F) and consistent coaching were **more likely to report higher confidence in using TIE skills in practice** compared to agencies with heavy leadership participation in coaching (i.e., Site G and Site C).

KEY TAKEAWAYS FROM COACHING FEEDBACK SURVEY (FEBRUARY – JUNE 2025)
**Strategic Planning & Structure**<br>*"The session assisted in our strategic planning of the DEIB Committee."* - Agency Senior Leader<br><br>*" (How) important (it is) to use structure to make sure goals are clear and steps are moving forward."* - Agency Senior Leader<br><br>**Enhanced Communication & Transparency**<br>*"Clear lines of communication need to be established between departments"* - Agency Senior Leader<br><br>**Cross-Site Engagement**<br>*"Creating space to hear other perspectives and experiences"* - Agency Senior Leader<br><br>*"I learned to ask questions in order to learn more about what people/staff are experiencing outside of the office"* - Agency Senior Leader<br><br>*"(It was) informative and learning to connect with (other) employees"* - Agency Senior Leader<br><br>**Self-Awareness & Care**<br>*"I am able to identify when my window of tolerance is being challenged, and ask for time to regroup"* - Direct-care Staff<br><br>*"I learned to be more mindful about assessing my state of mind and how to step in to my brave zone"* - Direct-care Staff<br><br>*"How to pause and reflect about what is really within my control and how I can influence higher levels of leadership, as well as model for my team"* - Agency Senior Leader

Specific Highlights:

- **Agency Senior Leadership accounted for ~70% of total sessions attended**, receiving a heavier "dose" of support. This suggests that **coaching may have skewed more towards organizational strategy (e.g., change initiative) and supervisory skills, rather than frontline application of skills with constituents.**
- Coaching participation varies widely by agency. Sites F & G accounted for the majority of engagement (based on number of participants and sessions attended), while Sites C & H **participated in 6 or less sessions.**
- This uneven distribution suggests that while all agencies were exposed to coaching, **some received a higher 'dose' of tailored support than others**.
- Agencies with higher coaching engagement are **likely better positioned to sustain TIE practices**, whereas lower-engaged agencies may require additional support to sustain efforts.

Training of the Trainer (ToT) Results

Participant Demographics:

- **15 total participants** in training sessions, with 5 Senior Leaders and 10 Direct-care staff trained, the **complete opposite of coaching session participants.**
- Four newer agencies engaged who **weren't previously involved in coaching or learning sessions**
- The majority of training participants **identified as women** in both pre- and post-surveys.
- **Broad racial/ethnic representation**, with Black or African American, Hispanic/Latino/Latinx, and White participants as the dominant groups.

Highlights from ToT Evaluation Report[11]:

- **High agreement** that training content (trauma-informed care, resilience, racial equity, SEEK tool, "Window of Tolerance") was directly applicable to participants' day-to-day work.
- **Significant increases in participants' confidence** to identify trauma responses, manage stress while facilitating, address problematic participant behaviors, and engage groups through interactive activities immediately following training.
- Role plays, scenario-based learning, and peer discussions were **consistently rated as most effective training methods.**
- Post-training, participants felt **more equipped and motivated to apply TIE concepts within their own departments.**
- Departments began integrating content into internal training efforts **soon after the ToT, demonstrating scalability.**

[11] Betz, M. G. (2025). City Learning Collaborative: Promoting Trauma-Informed & Equitable Approaches and Workforce Well-Being 2025. Capacity Building & Training Initiative. Boston Public Health Commission (BPHC).

Effectiveness & Implementation

In reviewing the following domain, please consider the following guiding questions:

- Which outcomes (organizational readiness and capacity, ProQOL, self-care, AFAS) showed the greatest improvement, and what do you think explains these shifts?
- How consistently was the LC content delivered across sites, and what adaptations do you believe were most helpful?
- What additional supports (e.g., coaching, role-specific groups) would make LC content more relevant to daily practice?

Please note: The full Readiness & Capacity report can be found beginning on page 31 of the appendix.

Organizational Level Outcomes:
Readiness and Capacity (R&C)

Organizational Readiness & Capacity	Baseline	Midline	Endline
**General Capacity**	**5.14**	**4.94**	**5.68**
Culture	5.78	5.92	5.68
Climate	4.84	4.69	5.01
Structure	4.39	4.30	4.54
Org. Innovativeness	5.22	4.73	5.04
Resource Utilization	4.44	3.80	4.25
Leadership	6.13	5.72	5.73
Org. Relationships	4.56	4.40	6.00
Staff Capacities	5.00	5.25	1.51

1 = Strongly Disagree	2 = Disagree	3 = Slightly Disagree	4 = Neither Agree nor Disagree	5 = Slightly Agree	6 = Agree	7 = Strongly Agree

Specific Highlights:

- **General organizational capacity improved moderately** across the collaborative, with notable strengths in culture, climate, structure, and cross-site relationships.
- **Leadership perceptions remained relatively stable**, suggesting consistent support across time, while resource utilization dipped sharply at midline before returning closer to baseline at endline.
- **Staff capacities declined steeply by endline**, highlighting a critical gap between organizational-level improvements and frontline staff's confidence in applying TIE skills. This may also reflect workforce fatigue or burnout.

Trauma Informed Organizational Assessment (TIOA)

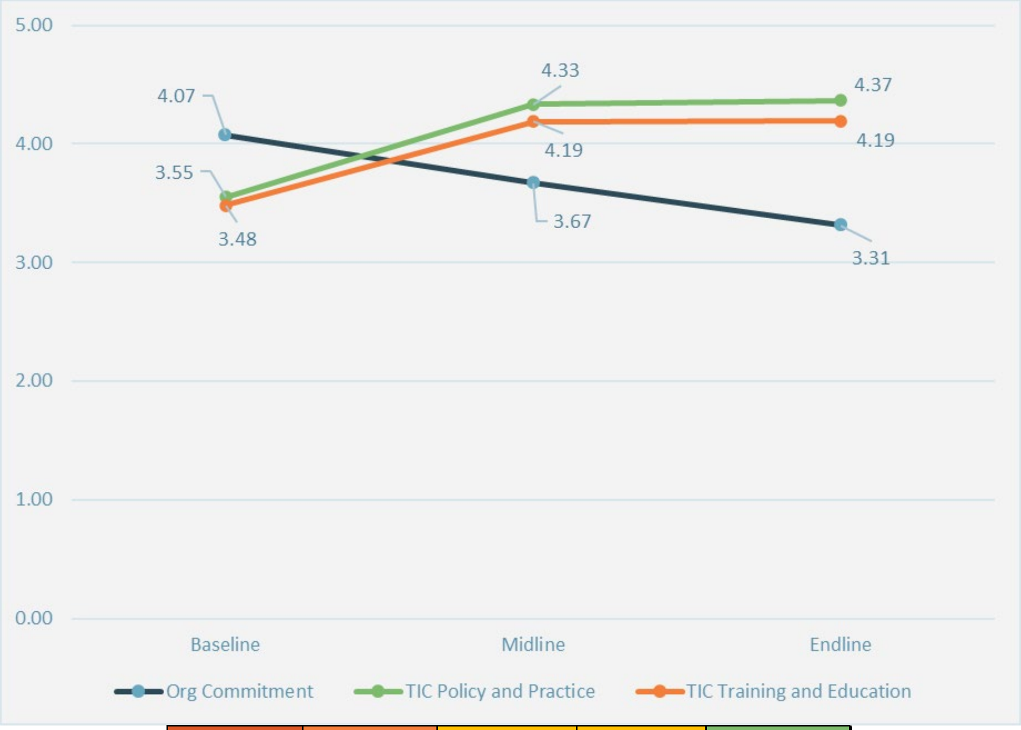

1 = No Progress	2 = Little Progress	3 = Some Progress	4 = Process in place, not sustained	5 = Process complete and sustained

Specific Highlights:

- **Organizational commitment scores declined**, pointing to competing priorities and challenges in sustaining high-level buy-in over time.
- **Policy, procedure, and practice scores showed steady improvement**, with gains in document review, supervision, performance review, and TIC training, suggesting strengthened infrastructure that may endure beyond the collaborative.
- **TIC training and education improved and stabilized**, with evidence that both training and skill development increased across the collaborative, though the pace of progress varied.

Overall, results *paint* to a *"*mixed*"* picture: while structural and policy-level progress is evident, sustaining leadership commitment and ensuring frontline staff feel equipped and supported remain key challenges for Cohort 2 and future implementation.

Sustainability: "This training is really great for us. Now, how do we keep it going when priorities keep shifting?" (Senior Leader)

Leadership Commitment: "Working really with, like our director, to make sure this isn't just a training but something we're actually doing day-to-day." (Senior Leader)

Barriers: "It was really hard to narrow down what we wanted to focus on (Change Initiative) because everyone had different priorities." (ToT Trainer)

Individual Level Outcomes

Professional Quality of Life

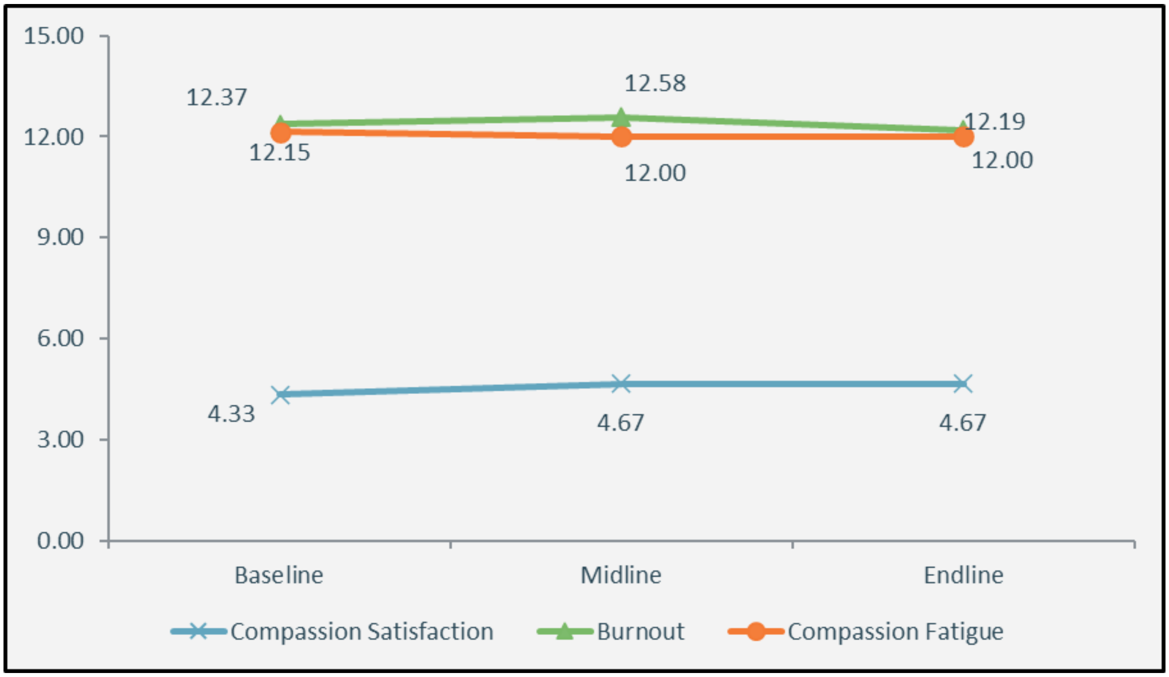

Low - 1	Low - 1	Low - 1
Medium - 11	Medium - 7	Medium - 5
High - 14+	High - 9+	High - 6+

Specific Highlights:

- **Compassion satisfaction remained low,** indicating limited increases in meaning or rewards from work.
- **Burnout stayed high**, pointing to persistent risks from workload and resource gaps.
- **Compassion fatigue remained high**, suggesting continued strain from constituents or other work-related stressors.

***"We care about the people that work here and listen to them, but there's only so much you can do with the workload."* (Direct-care Staff)**

"It (Learning Collaborative) gave me the tools to feel like I could be a bit more present, even when things are stressful." (Senior Leader)

Self-Care Assessment (SCA)

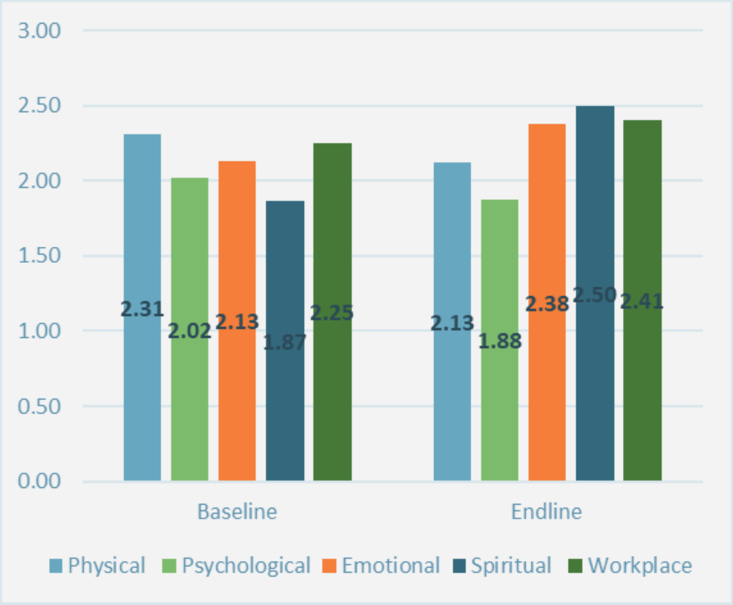

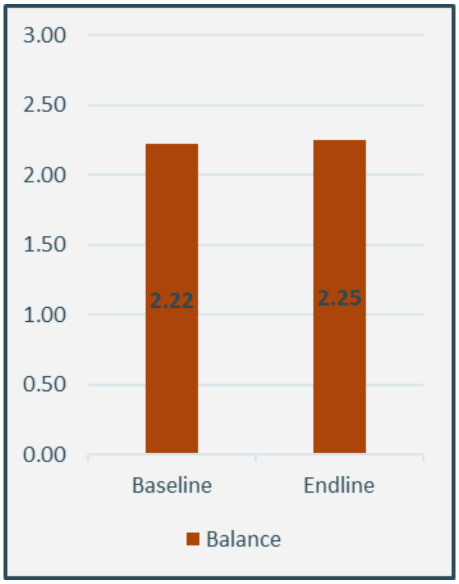

0 = Never occurred	1 = Rarely	2 = Occasionally	3 = Frequently

Specific Highlights:

- **Physical and workplace self-care stayed in the occasionally – frequently range**, showing positive habits that may continue to buffer burnout post Learning Collaborative.
- **Emotional and spiritual self-care improved from baseline**, moving closer to the occasionally–frequently range, suggesting increased reflective and emotional supports.
- **Balance was rated around occasionally at both timepoints,** with a slight rise since baseline, pointing to small but measurable improvements in maintaining equilibrium across work, family, and relationships.

"Oh, so on a personal level, I probably say I do my best, but it's hard to find balance when demands keep piling up." (Direct-care Staff)

Similar to organization-level outcomes, individual level outcomes paint a "mixed" picture. While staff demonstrated modest gains in self-care strategies and overall "balance", low compassion satisfaction and sustained levels of burnout and compassion fatigue point to ongoing risks across sites.

These findings suggest that current support may not be sufficiently addressing the full scope of emotional demands of the work. However, this offers an opportunity to expand Cohort 2 content and support.

AFAS Scale – "Overall perceptions of the TIE Learning Collaborative"

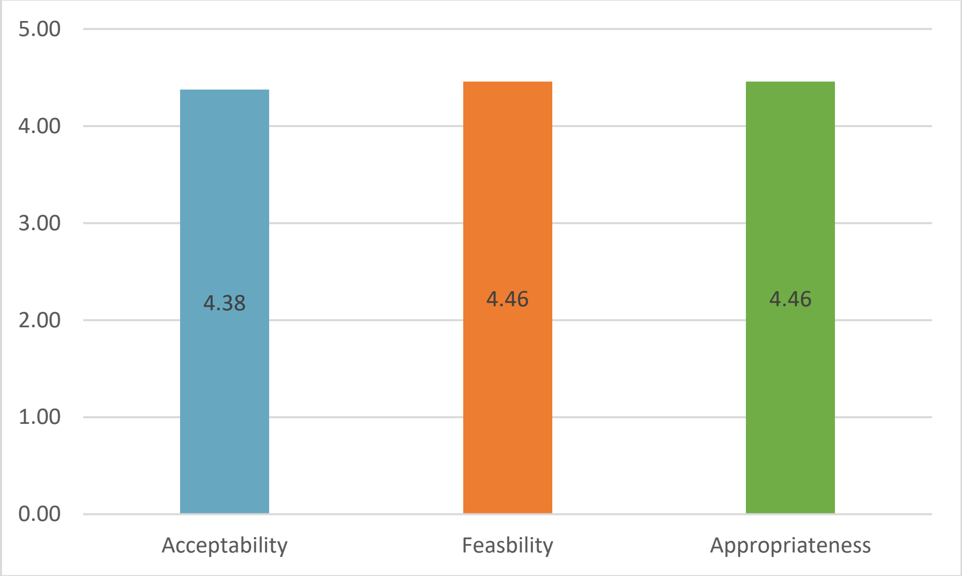

1 = Not at all	2	3 = Moderately	4 =	5 = Extremely

Specific Highlights:

- **Acceptability** scored high, suggesting participants were extremely satisfied with training content, organization, and relevance.
- **Feasibility** scored high, indicating practices were seen as very compatible with existing workflows and participant skillsets.
- **Appropriateness** scored high, reflecting strong alignment between practices and agency missions, service delivery approaches, and client needs.

Overall: Unlike the more "mixed" results at the individual level, **AFAS findings show consistently strong perceptions of the initiative.** Participants found the Learning Collaborative highly acceptable, feasible, and appropriate, underscoring its strong design and fit with both practice and organizational context.

Endline Focus Group Findings

KEY TAKEAWAYS FROM FOCUS GROUPS – LEARNING COLLABORATIVE<br>AUGUST 2024
By the close of the Learning Collaborative, participants reported that while initial sessions largely reinforced knowledge they already held, **the later coaching and peer-to-peer discussions provided the greatest value**. Many highlighted that having protected space to share challenges and see how other sites applied skills in practice deepened their learning and relevance.<br><br>*"I really appreciated working with the facilitators. They were down to earth and conveyed the material in a way that was easy to learn from. They encouraged participation and made me feel comfortable to share and engage during the sessions, which I really valued."*<br>*– Direct-care staff*
Participants endorsed the following topics and skills from the Learning Collaborative that they felt were most relevant to current practice at endline, including:<br>• Locus of Control<br>• Trauma-Informed Supervision and Feedback<br>• Trauma-Informed "Constituent-Facing" Practices<br>• Conflict Resolution and Problem-Solving Approaches

Participants across roles highlighted both drivers (facilitators) and challenges (barriers) to carrying forward their change initiatives beyond the Learning Collaborative. While staff valued **practical tools and peer learning as critical support**, they also described **real barriers such as shifting priorities and staff capacity**. The table below summarizes these factors identified from focus group findings.

KEY TAKEAWAYS FROM FOCUS GROUPS – SUSTAINABILITY DRIVERS<br>AUGUST 2025	
**FACILITATORS**	**BARRIERS**
**Practical tools** (e.g., locus of control, trauma-informed feedback) that staff could apply directly in their work and personal life.	**Heavy workloads and administrative demand** reduced capacity to integrate new practices sustainably.
**Peer learning and cross-site collaboration** reinforced confidence and normalized challenges across agencies and roles.	Participants shared that **organizational changes and competing priorities** made it difficult to sustain buy-in.
**TIC/Equity-centered values** created shared purpose and team motivation to continue efforts.	**Direct-care staff felt primarily responsible** for carrying out changes without adequate organizational support.
**ToT Training** built confidence by preparing trainers to embed practices within their organizations.	**Lack of formalized protocols** to embed skills across agencies presented limited follow-through.

Additional details on how participant perspectives evolved from July 2024 through August 2025 are included in the Appendix (see Table 2: Change Over Time in Participant Perspectives by Role).

Throughout the Implementation Phase, BPHC leadership made notable adaptations to enhance participant experience. Though not an exhaustive list, some of those adaptations include the following:

- **Developed key resources** (e.g., The Mental Health Toolkit, Critical Incident Protocol, Goal Setting and Readiness Tool, Prioritization Matrix Tool, Conflict Resolution Resources, PDSA Worksheet) to enhance agency planning and implementation efforts.
- Offered virtual and in-person Learning and Coaching sessions to **enhance participant engagement across sites**.
- Developed and distributed **completion certificates to participants** after their concluding Cohort 1 activities.
- And many more!

Setting the Scope of Findings:

- **Sample size and generalizability**: Survey response rates averaged **~54%.** While responses provide valuable insights into participant experiences and outcomes, the small sample size (~15 or less people) limits statistical power. Findings should therefore be interpreted as directional trends rather than statistically significant results.

- **Representation of participants**: The average implementation team was **~2 persons**, one senior leader and one direct care staff. While this structure elevated program champions, it may not fully represent the perspectives of the broader workforce at each site.

- **Potential bias (Hawthorne / desirability effect)**: Feedback may be skewed toward participants with higher initial buy-in, either because they were more likely to complete surveys and participate in focus groups or because they wanted to represent well to BPHC. This may result in overly positive perceptions compared to staff who were less engaged.

- **Timeframe**: Results reflect outcomes measured within the implementation period of Cohort 1. Long-term agency sustainability and system-level impacts could not be assessed within this timeframe.

Despite these limitations, the findings provide meaningful insights into how Cohort 1 participants engaged with and experienced the LC. While not statistically generalizable, the data highlight clear trends and practical lessons that can guide enhancements for Cohort 2 and inform future efforts.

Maintenance

In reviewing the following domain, please consider the following guiding questions:

- What structures (participation requirements, evaluation processes, etc.) are needed to enhance efforts in Cohort 2?
- Which lessons from Cohort 1 should be formalized into Cohort 2 design to reduce reliance on individual champions?

The Maintenance section focuses on how lessons from Cohort 1 can be carried forward to strengthen Cohort 2 and ensure long-term sustainability of the Learning Collaborative (LC). While Cohort 1 demonstrated important successes, including valued cross-site learning and practical tools applied in practice - participant and evaluation data also highlighted gaps in representation, consistency, and direct participant feedback. The analysis below synthesizes these findings into a SWOT framework[12] to guide actionable improvements in both program quality and evaluation systems.

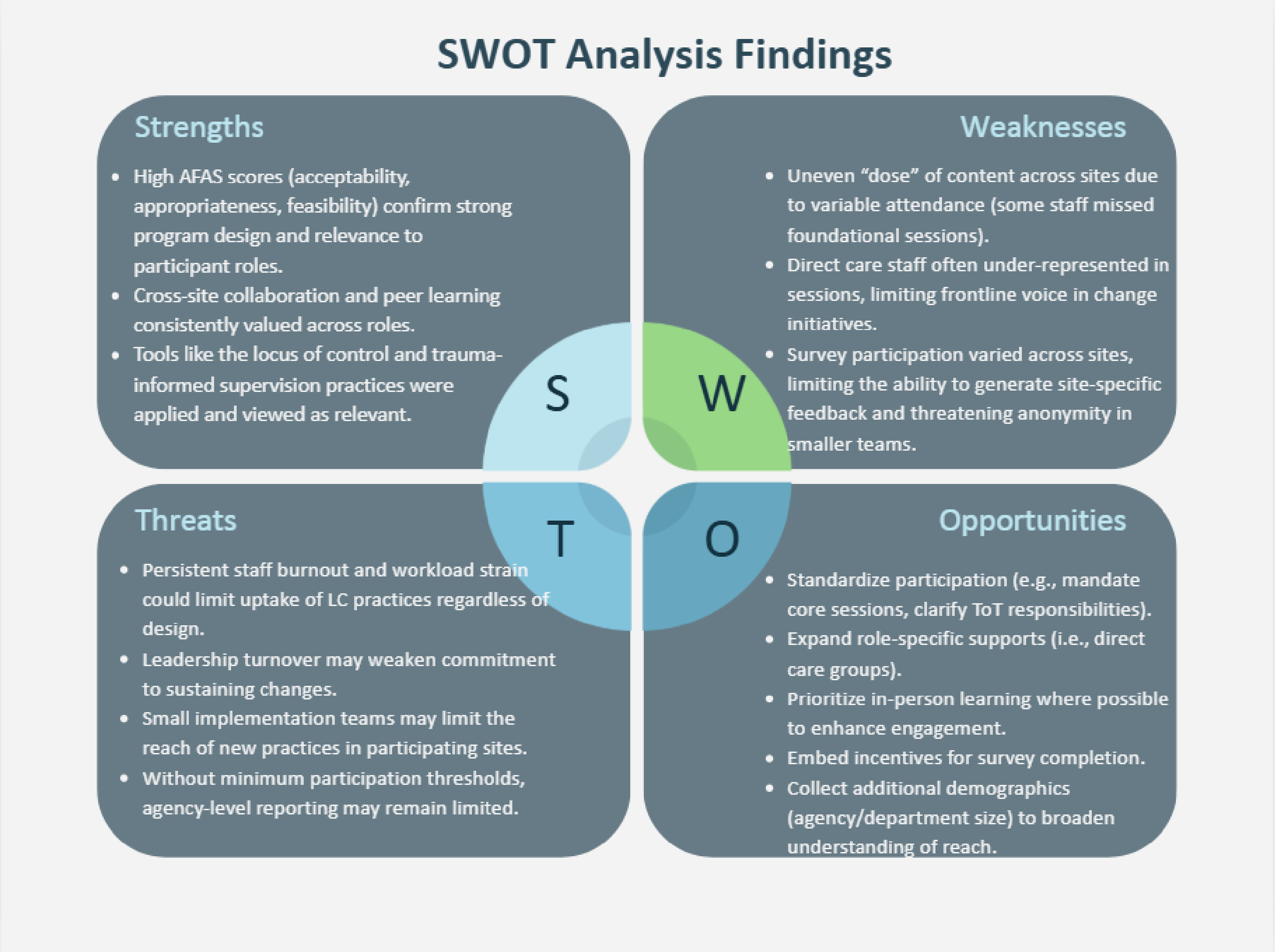

[12] Puyt, R. W., Lie, F. B., & Wilderom, C. P. M. (2023). The Origins of SWOT Analysis. Long Range Planning, 56(3), 102304. https://doi.org/10.1016/j.lrp.2023.102304

Key Recommendations:

Program Quality Improvement

- **Increase representation and balance:** Encourage sites to include a broader mix of staff in the LC, with particular attention to direct care staff. Consider creating role-specific spaces (e.g., support groups for direct care staff) to ensure their perspectives are elevated without being overshadowed by leadership.
- **Standardize foundational content:** Ensure all sites receive the same "dose" of core training by making learning sessions mandatory. Where possible, prioritize in-person sessions over virtual, given their stronger impact on engagement.
- **Strengthen cross-site collaboration:** Reinforce the LC's role as a peer-learning space by building in structured opportunities for cross-site exchange. Where helpful, organize discussions by role type to maximize relevance and openness.
- **Clarify ToT expectations:** Provide clear guidelines that ToTs should attend Learning Sessions to fully grasp the scope of content they will be asked to train and reinforce within their agencies.

Evaluation Quality Improvement

- **Collect evaluations in real time:** Build time into sessions for participants to complete evaluations (including paper copies when in person) and require submission before signing off virtually.
- **Ensure adequate representation for site-level feedback:** Set a minimum participation threshold (e.g., 3–4 respondents per site) so agency-specific reports can be shared without compromising anonymity. This is particularly important for direct care staff, who may feel most vulnerable providing feedback.
- **Consider incentives:** Explore low-burden incentives to encourage timely and complete survey responses.
- **Capture demographic information:** Collect additional demographics (e.g., role, agency size, etc.) to broaden the picture of reach and ensure results reflect diverse perspectives across sites.

To enhance the Learning Collaborative for Cohort 2, program leadership should focus on strengthening both program quality and evaluation systems. On the program side, this means ensuring balanced representation, standardizing foundational training, clarifying ToT roles, and expanding structured opportunities for peer exchange. On the evaluation side, priorities include collecting data in real time, ensuring adequate representation for site-level feedback, incentivizing survey participation, and capturing demographic and agency-level data to better understand equity and reach.

Together, these recommendations provide a pathway for Cohort 2 to build on the strengths of Cohort 1 while addressing gaps in representation, consistency, and evaluation. By enhancing both program delivery and evaluation systems, the Learning Collaborative can deepen its impact, generate more actionable data, and ensure sustainable change across participating sites. These enhancements position Cohort 2 not only to refine the implementation of trauma-informed practices, but also to provide clearer evidence of impact that can guide long-term sustainability.

Appendix

Learning Session Training Feedback

Scale				
Very Little	Little	Some	A good bit	A great deal
1	2	3	4	5

**Please choose one answer for each question below that best represents your response to each statement about the learning goals of the training. (n=9)**	**Before**	**After**
I understood how stress levels can impact my work and quality of life (e.g., window of stress tolerance)	4.22	4.89
I understood the importance of self-care and self-preservation in my work.	4.56	4.78
I understood the difference between trauma informed and trauma specific approaches	3.33	4.11
I understood TIE principles and how to apply them in practice.	3.44	4.11
I could define trauma, resilience, and equity.	3.67	4.78
I understood how a trauma informed, and equitable approach applies to my work.	3.89	4.67
How much did you learn as a result of this learning session?	4.33	

**Learning Session 2: Please choose one answer for each question below that best represents your response to each statement about the learning goals of the training. (n=3)**	**Before**	**After**
I could identify at least 1 self-care and/or coping strategy to help manage workplace stress.	4.67	5.00
I could identify at least 1 individual and organizational sign of stress (e.g., burnout, compassion fatigue, etc.).	4.67	5.00
I could identify at least 1 sign of vicarious trauma in my workplace.	4.00	5.00
How much did you learn as a result of this learning session?	4.00	

**Learning Session 3: Please choose one answer for each question below that best represents your response to each statement about the learning goals of the training. (n=5)**	**Before**	**After**
I understood ways in which my values align with my current work and practice.	4.60	4.40
I could clearly articulate anti-racism efforts in the city of Boston.	3.80	4.20
How much did you learn as a result of this learning session?	4.20	

**Learning Session 4: Please choose one answer for each question below that best represents your response to each statement about the learning goals of the training. (n=1)**	**Before**	**After**
I could identify and discuss the top 3 signs of vicarious trauma.	3.00	4.00
I could identify and discuss the 4 elements of overwhelm and managing.	3.00	4.00
I had clearly defined tools and strategies responding to crisis within my role. (SEEK)	2.00	4.00
How much did you learn as a result of this learning session?	3.00	

Learning Session 5: Please choose one answer for each question below that best represents your response to each statement about the learning goals of the training. (n=2)	Before	After
I could identify and discuss at least 3 strategies to facilitate trauma informed conversations in my workplace.	2.50	4.50
I could identify and discuss the 3 stages of change.	4.00	4.50
How much did you learn as a result of this learning session?	5.00	

Learning Session 6: Please choose one answer for each question below that best represents your response to each statement about the learning goals of the training. (n=4)	Before	After
I could identify and discuss ways collected data can help inform decision making.	4.75	5.00
I could identify and discuss the role of coaching to support TIE practice and skills.	3.75	4.50
I could identify and discuss the components and utility of a critical incident protocol.	3.00	4.25
How much did you learn as a result of this learning session?	4.50	

Appendix Table 1. Coaching Takeaways

Key Takeaways from Coaching Sessions
The session assisted in our strategic planning of the DEIB Committee
(How) important (it is) to use structure to make sure goals are clear and steps are moving forward to reach them
Creating space to hear other perspectives and experiences
I learned to ask questions in order to learn more about what people/staff are experiencing outside of the office that might be impacting their work.
I am able to identify when my window of tolerance is being challenged and ask for time to regroup.
How to pause and reflect about what is really within my control and how I can influence higher levels of leadership, as well as model for my team
TRANSPARENCY/ENGAGEMENT/LIFE SKILLS
I learned to be more mindful about assessing my state of mind and how to step into my brave zone
How to best lead session while transmitting compassion and understanding
Self-care
The window of tolerance
Clear lines of communication need to be established between departments
informative and learning to connect with employees

Cross Site Readiness Report

Readiness and Capacity – Endline Report (Expanded)

Assessments Administered:

- Readiness Diagnostic Tool[13]
- Trauma Informed Organizational Assessment (TIOA)[14]
- Professional Quality of Life (ProQOL) Scale[15]
- Self-Care Assessment (SCA)[16]
- Acceptability, Feasibility, and Appropriateness (AFAS) Scale[17]

Respondents:

- 8 staff (Direct-care staff, supervisors, and senior leaders ONLY)

Disclaimer: This report is based on data collected from participating agencies. Due to a low response rate, results specific to individual agencies cannot be shared to maintain confidentiality and data reliability. Additionally, findings in this cross-site analysis should be interpreted with caution, as they do not meet thresholds for statistical significance and are intended to provide general trends rather than definitive conclusions.

---

[13] Scaccia, J. P., Cook, B. S., Lamont, A., Wandersman, A., Castellow, J., Katz, J., & Beidas, R. S. (2015). A practical implementation science heuristic for organizational readiness: R = MC2. Journal of community psychology, 43(4), 484-501.

[14] Cellarius, K., Davis, M., Tuttle, A., daRosa, C. (2023) PSU/TIO Trauma Informed Care Implementation Assessment Instrument, v1.2. Portland, OR: Portland State University.

[15] Stamm, B. H. (1995). Professional Quality of Life Scale (PROQOL) and Galiana, L., Oliver, A., Arena, F., De Simone, G., Tomás, J. M., Vidal-Blanco, G., Muñoz-Martínez, I., & Sansó, N. (2020). Development and validation of the Short Professional Quality of Life Scale based on versions IV and V of the Professional Quality of Life Scale. Health and quality of life outcomes, 18(1), 364. https://doi.org/10.1186/s12955-020-01618-3

[16] Adapted from Saakvitne, Pearlman, & Staff of TSI/CAAP (1996). Transforming the pain: A workbook on vicarious traumatizaton. Norton.

[17] Cho, E., Lyon, A. R., Tugendrajch, S. K., Marriott, B. R., & Hawley, K. M. (2022). Assessing provider perceptions of training: Initial evaluation of the Acceptability, Feasibility, and Appropriateness Scale. Implementation Research and Practice, 3, Article 26334895221086269. https://doi.org/10.1177/26334895221086269

Organizational Level Outcomes

<u>Readiness and Capacity (R&C)</u>

Goal:

- To get a sense of your organization's ongoing readiness and capacity for supporting the implementation of skills learned from the TIE learning collaborative.
- To gauge ongoing needs of organizations in order to support.

Administration and Use:

- Completed 3 times throughout the Learning Collaborative
- Completed individually to reflect how <u>organizations</u> are performing across the LC
- Results integrated into coaching throughout Learning Collaborative
- Higher scores indicate greater readiness and capacity

Organizational Readiness & Capacity				Baseline	Midline	Endline
**General Capacity**				**5.14**	**4.94**	**5.68**
Culture				5.78	5.92	5.68
Climate				4.84	4.69	5.01
Structure				4.39	4.30	4.54
Org. Innovativeness				5.22	4.73	5.04
Resource Utilization				4.44	3.80	4.25
Leadership				6.13	5.72	5.73
Org. Relationships				4.56	4.40	6.00
Staff Capacities				5.00	5.25	1.51
1 = Strongly Disagree	2 = Disagree	3 = Slightly Disagree	4 = Neither Agree nor Disagree	5 = Slightly Agree	6 = Agree	7 = Strongly Agree

Highlights:

- **Organizational general capacity improved moderately** across the Learning Collaborative. Organizational culture, climate, structure, and cross-site relationships are strengths.
- Throughout the Learning Collaborative, **perceptions of organizational leadership have remained stable** while resource utilization showed a sharp decline at midline and return in baseline results at the end of implementation.
- **Staff capacities experienced a sharp decline** through Endline assessment, signaling a potential gap between organizational progress and frontline staff confidence in applying TIE skills. Additionally, there may be experience of burnout from participation in the Learning Collaborative.

Trauma Informed Organizational Assessment (TIOA)

Goal:

- To understand how organizational staff adopt key elements of trauma informed care (TIC) in order to inform implementation and Learning Collaborative outcomes.

Administration and Use:

- Completed 3 times throughout the Learning Collaborative
- Completed individually to reflect how organizations are performing across the LC
- Results integrated into coaching throughout Learning Collaborative
- Higher scores indicate greater readiness and capacity

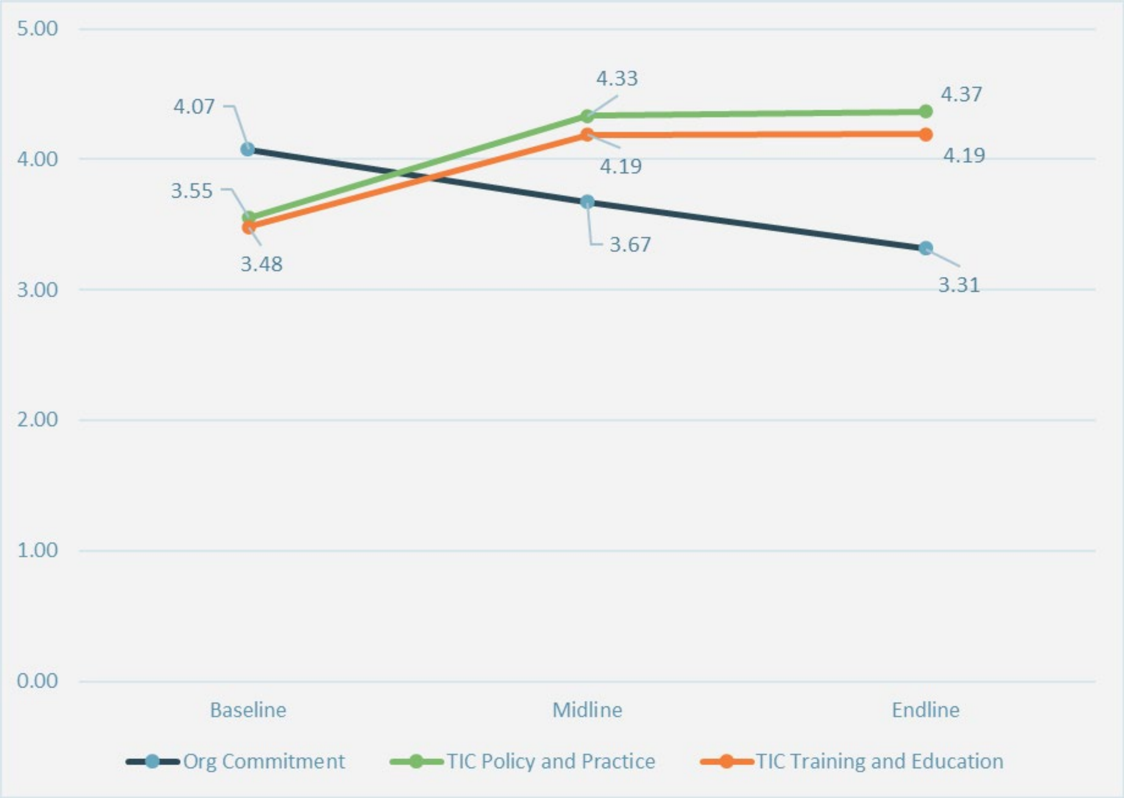

1 = No Progress	2 = Little Progress	3 = Some Progress	4 = Process in place, not sustained	5 = Process complete and sustained

Highlights:

- **Organizational commitment scores have declined since baseline**, indicating that there may be competing priorities and challenges in sustaining high-level buy-in within organizations.
- **Policy, Procedure, and Practice scores continue to improve since Baseline**. Notable gains in document review, supervision, performance review, and TIC training contribute to this improvement, suggesting that concrete structures were strengthened over time and may be sustained.
- **TIC Training and Education improved overall** and remained consistent by Endline. TIC Training and TIC Skill domains have seen notable improvement across the TIE Learning Collaborative,

Individual Level Outcomes

Professional Quality of Life

Goal:

- To understand perceptions of professional quality of life in relation to one's work as a direct service provider. This assessment targets 3 areas: compassion satisfaction, provider burnout, and compassion fatigue

Administration and Use:

- Completed 3 times throughout the Learning Collaborative
- Completed Individually by all members of the Implementation Team
- Results integrated into coaching throughout Learning Collaborative
- Scores out of 15 across domains, with different “cut-offs” for severity

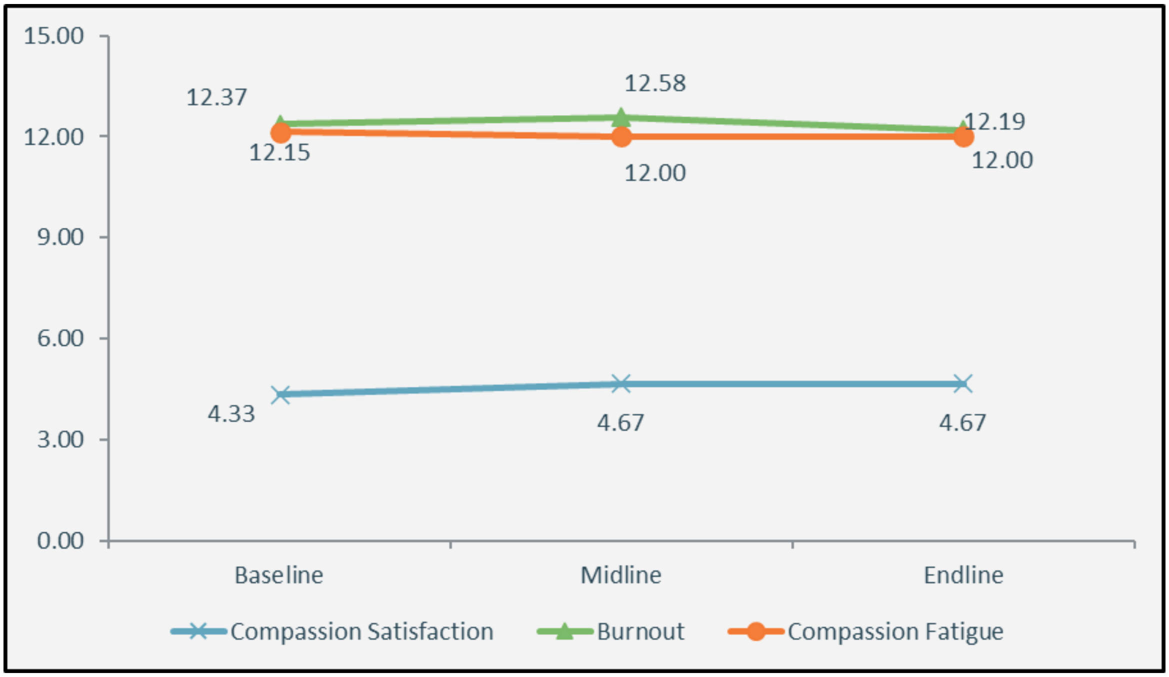

Low - 1	Low - 1	Low - 1
Medium - 11	Medium - 7	Medium - 5
High - 14+	High - 9+	High - 6+

Highlights:

- **Compassion satisfaction scores remained low** across the Learning Collaborative, suggesting that participants did not experience notable increases in fulfillment or rewards from their work throughout

the course of activities. This suggests organizations may need to focus more intentionally on strategies that strengthen meaning-making, recognition, and opportunities for positive feedback.

- **Burnout scores remained high**, signaling a consistent risk of compassion fatigue. Elevated burnout often reflects a mismatch between job demands and available resources. Strategies to address this include workload adjustments, expanded supervision and peer support, and better alignment of administrative demands with available capacity.
- **Compassion fatigue also remained high**, underscoring that exposure to client-related stressors continues to impact staff. Interventions focused on wellness resources, reflective practice, and embedding trauma-informed supports into organizational culture are needed to mitigate long-term risks.

Key

- **Compassion Satisfaction (CS)** refers to the pleasure derived from helping, affection from colleagues and finding meaningful fulfillment in work. Compassion satisfaction is the direct opposite of compassion fatigue and can protect workers from burnout and secondary traumatic stress.
- **Compassion Fatigue (CF)** refers to the continuing stress of meeting the often-overwhelming needs of families. This area can impact the direct service provider (job satisfaction, overall well-being) and workplace environment (e.g., decreasing productivity, quality of work, agency turnover).
- **Burnout (BO)** refers to feeling emotionally depleted or drained and can result after a period of long-term, work-related stress. Burnout can be categorized by emotional exhaustion, cynicism and feelings of inefficacy.

Self-Care Assessment

Goal:

- To understand current self-care practices across the learning collaborative and possible areas to minimize burnout and compassion fatigue.

Administration and Use:

- Completed 2 times throughout the Learning Collaborative (Baseline and Endline)
- Completed individually by all members of the Implementation Team

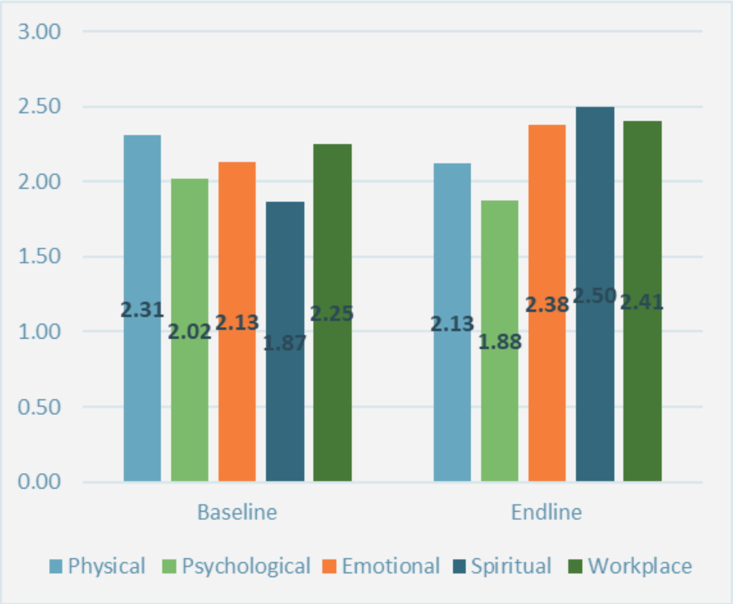

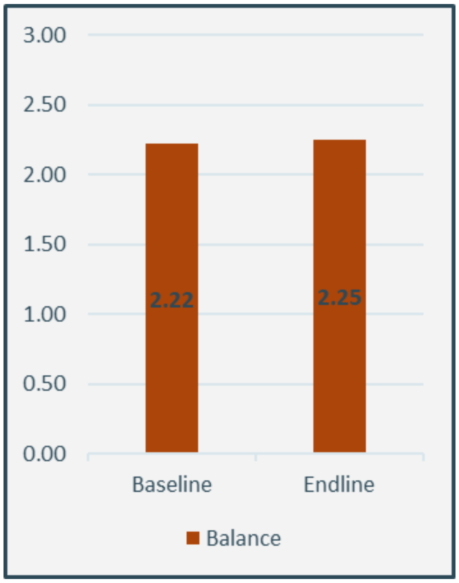

0 = Never occurred	1 = Rarely	2 = Occasionally	3 = Frequently

Highlights:

- **Physical and workplace self-care** scores were moderately high, suggesting that staff are engaging in activities that support personal and professional wellbeing. Continued attention to these areas could help reduce burnout and sustain resilience.
- **Spiritual and psychological self-care** scores were more moderate, indicating opportunities to strengthen support such as reflective practice, supervision content, or team-based activities that promote emotional and spiritual wellbeing.
- **Overall balance was rated around occasionally at both timepoints,** with a slight rise since baseline, pointing to small but measurable improvements in maintaining equilibrium across work, family, and relationships.

Key

- **Physical Self Care** refers to personal activities (e.g., regular eating, exercise, getting enough sleep, etc.).
- **Psychological Self Care** refers to seeking activities that decrease stress in one's life (e.g., journaling, letting others know different aspects of you, receiving from others, etc).
- **Emotional Self Care** refers to activities such as spending time with loved ones, self-affirmations, identifying comforting activities, objects, people, or places and seeking them to bring joy.
- **Spiritual Self Care** refers to finding spiritual connection or community, cherishing optimism and hope, openness to "not knowing", and identifying what's meaningful and it's place in your life.
- **Workplace/Professional Self Care** refers to activities that create space for rest during a workday (breaks or lunch), identifying projects or tasks that are exciting or fulfilling, having a workplace environment that is comfortable, and regular supervising and/or consultation.
- Overall, **Balance** refers to perceptions of achieving equity among work, family, relationships, play, and rest.

Acceptability, Feasibility, and Appropriateness Scale (AFAS)

Goal:

- To understand current perceptions of participant ability to use the practices and resources provided throughout the Learning Collaborative.

Administration and Use:

- Completed 1 time throughout the Learning Collaborative (at Endline)
- Completed individually by all members of the Implementation Team

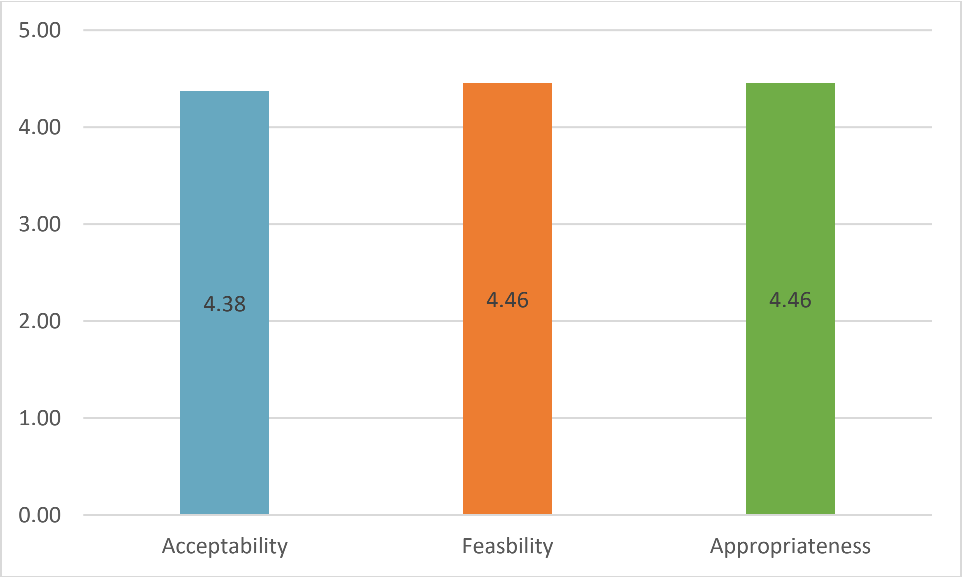

**1 = Not at all**	**2**	**3 = Moderately**	**4 =**	**5 = Extremely**

Highlights:

- **Acceptability scored high**, suggesting participants were not only satisfied with the training content but also found the structure and delivery approachable and engaging. This suggests that the Learning Collaborative successfully met expectations for relevance and quality, creating buy-in that can serve as a foundation for sustaining trauma-informed and equitable practices.
- **Feasibility scored high**, indicating practices were seen as very compatible with agency realities, workflows, and skillsets. The strong alignment with existing skillsets and organizational structures suggests that the TIE approach was seen as actionable rather than aspirational, increasing the likelihood of continued use beyond the formal collaborative period.
- **Appropriateness scored high**, reflecting strong alignment between practices and agency missions, service delivery approaches, and client needs. This finding highlights that the collaborative was not viewed as a "one-size-fits-all" program, but rather as a tailored initiative that resonated with both staff values and organizational priorities.

Key

- **Acceptability** refers to how much participants liked and were satisfied with the training and the practices it covered. It includes whether the content felt useful, whether the training was well-organized, and whether the presenters were credible. For example, "*Did people find the training worthwhile and comfortable to engage in?*"
- **Feasibility** captures how realistic and practical the practices from the training seemed to participants in their everyday work. It looks at whether the strategies fit into normal workflows, schedules, and available resources. For example, "*Can staff actually use these practices in their setting without major barriers?"*
- **Appropriateness** is about fit of the program. It measures whether the practices covered felt relevant to the agency's mission, the staff's professional role, and the needs of the clients they serve. For example, "*Does the training content make sense for this agency, these staff, and their communities?*"

Appendix Table 2. Change Over Time in Participant Perspectives by Role (2024–2025)

Role	2024 Findings	2025 Findings
**Direct-care Staff**	• Valued TIC principles and tools (e.g., locus of control) but much of the content felt familiar<br>• Wanted more applied discussion.<br>• Desired critical incident protocols, more opportunities for dialogue, and coaching to help "ground" content in daily practice.	• Reported LC content as directly relevant to work; cited locus of control as a coping support.<br>• Highlighted challenges balancing heavy workloads with applying new practices; requested more structured self-care supports and ongoing supervisor reinforcement.
**Supervisors / Mid-Level Leaders**	• Identified as a bridge between staff, community, and senior leadership.<br>• Requested trauma-informed feedback strategies, conflict resolution approaches, and support navigating organizational dynamics.	• Emphasized the importance of supervision as a sustainability driver; valued reflective practice and coaching strategies.<br>• Noted the ongoing burden of carrying change without consistent top-down support.
**Senior Leaders (SL)**	• Recognized value of TIC foundation but acknowledged competing priorities and turnover as barriers.<br>• Expressed need for sustainability planning, stronger organizational protocols, and leadership transparency.	• Reiterated importance of leadership consistency and follow-through.<br>• Highlighted need for protected time and formalized structures (policy/supervision) to embed practices and sustain change.
**ToT Trainers**	(Not applicable in July/Aug focus groups)	• Reported confidence gains from ToT training; emphasized need for continued practice, structured feedback, and opportunities to embed skills across their agencies.

Reference List

Cho, E., Lyon, A. R., Tugendriach, S. K., Marriott, B. R., & Hawley, K. M. (2022). Initial evaluation of the Acceptability, Feasibility, and Appropriateness Scale (AFAS): A provider-report measure of immediate implementation outcomes. *[Journal Name]*. This study examined the factor structure of the AFAS, showing a three-factor model (acceptability, feasibility, appropriateness), with good fit (RMSEA = .058, CFI = .990, TLI = .987) and high reliability (Cronbach's alpha = .86–.91) Semantic Scholar+6NACHC+6PubMed+6Taylor & Francis Online+3PubMed+3ResearchGate+3.

Feldstein, A. C., & Glasgow, R. E. (2008). A practical, robust implementation and sustainability model (PRISM) for integrating research findings into practice. *The Joint Commission Journal on Quality and Patient Safety, 34*(4), 228–243. https://doi.org/10.1016/S1553-7250(08)34030-6 ScienceDirect+9PubMed+9NACHC+9.

Galiana, L., Arena, F., Oliver, A., Sansó, N., & Benito, E. (2020). Compassion satisfaction, compassion fatigue, and burnout in Spain: Validation of the Professional Quality of Life Scale (ProQOL). *Health and Quality of Life Outcomes, 18*(1), 1–11. https://doi.org/10.1186/s12955-020-01318-9

Glasgow, R.E., Trinkley, K.E., Ford, B. *et al.* (2024). The Application and Evolution of the Practical, Robust Implementation and Sustainability Model (PRISM): History and Innovations. *Glob Implement Res Appl* **4**, 404–420. https://doi.org/10.1007/s43477-024-00134-6

Kowalski, C. P., Nevedal, A. L., Finley, E. P., Young, J. P., Lewinski, A. A., Midboe, A. M., & Hamilton, A. B. (2024). Planning for and Assessing Rigor in Rapid Qualitative Analysis (PARRQA): a consensus-based framework for designing, conducting, and reporting. *Implementation science : IS*, *19*(1), 71. https://doi.org/10.1186/s13012-024-01397-1

Rabin, B.A., Cakici, J., Golden, C.A. et al. (2022). A citation analysis and scoping systematic review of the operationalization of the Practical, Robust Implementation and Sustainability Model (PRISM). Implementation Sci 17, 62. https://doi.org/10.1186/s13012-022-01234-3

Saakvitne, K. W., Pearlman, L. A., & Staff of TSI/CAAP. (1996). *Transforming the pain: A workbook on vicarious traumatization.* W. W. Norton & Company.

Scaccia, J. P., Cook, B. S., Lamont, A., Wandersman, A., Castellow, J., Katz, J., & Beidas, R. S. (2015). A practical implementation science heuristic for organizational readiness: R = MC^2. *Journal of Community Psychology, 43*(4), 484–501. https://doi.org/10.1002/jcop.21698

Stamm, B. H. (2010). *The concise ProQOL manual* (2nd ed.). Pocatello, ID: ProQOL.org.

Trauma Informed Oregon & PSU Human Services Implementation Lab. (2023). *Trauma-Informed Care (TIC) Organizational Self-Assessment.* Portland State University.

Puyt, R. W., Lie, F. B., & Wilderom, C. P. M. (2023). The Origins of SWOT Analysis. Long Range Planning, 56(3), 102304. https://doi.org/10.1016/j.lrp.2023.102304

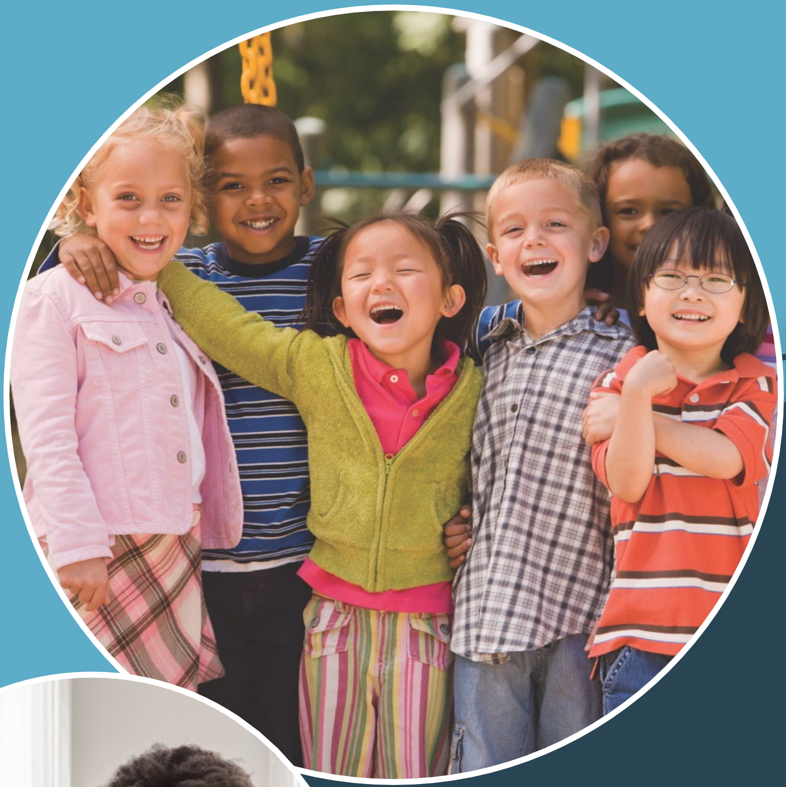

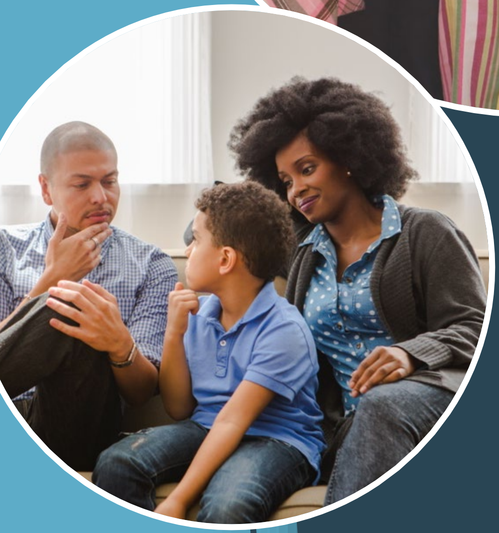

This report summarizes the evaluation results of the TIE Learning Collaborative (Cohort 1) hosted by The Boston Public Health Commission (BPHC). If you would like more information about the report or are interested in other program evaluation options, please contact qci@bakercenter.org.

Jessie Fitts, Ph.D.
Director of Clinical Implementation
jfitts@bakercenter.org

Sean Snyder, LCSW, DPA
Director of Implementation & Technical Assistance
ssnyder@bakercenter.org

THE BAKER CENTER
FOR CHILDREN AND FAMILIES

The Baker Center for Children & Families
53 Parker Hill Avenue, Boston, MA 02120 | Phone: (617) 232-8390
